# BCAA nitrogen flux in brown fat controls metabolic health independent of thermogenesis

**DOI:** 10.1016/j.cell.2024.03.030

**Published:** 2024-04-22

**Authors:** Anthony R.P. Verkerke, Dandan Wang, Naofumi Yoshida, Zachary H. Taxin, Xu Shi, Shuning Zheng, Yuka Li, Christopher Auger, Satoshi Oikawa, Jin-Seon Yook, Melia Granath-Panelo, Wentao He, Guo-Fang Zhang, Mami Matsushita, Masayuki Saito, Robert E Gerszten, Evanna L. Mills, Alexander S. Banks, Yasushi Ishihama, Phillip J. White, Robert W. McGarrah, Takeshi Yoneshiro, Shingo Kajimura

**Affiliations:** 1 Division of Endocrinology, Diabetes and Metabolism, Beth Israel Deaconess Medical Center and Harvard Medical School, and Howard Hughes Medical Institute, Boston, MA, USA; 2 Division of Cardiovascular Medicine, Beth Israel Deaconess Medical Center and Harvard Medical School, Boston, MA, USA; 3 Graduate School of Pharmaceutical Sciences, Kyoto University, Kyoto, Japan; 4 Duke Molecular Physiology Institute, Duke School of Medicine, Department of Medicine, Division of Endocrinology, Metabolism and Nutrition, Duke University, Durham, NC, USA; 5 Department of Nutrition, School of Nursing and Nutrition, Tenshi Collage, Sapporo, Japan; 6 Laboratory of Biochemistry, Faculty of Veterinary Medicine, Hokkaido University, Sapporo, Japan.; 7 Department of Cancer Immunology and Virology, Dana-Farber Cancer Institute, and Department of Immunology, Harvard Medical School, Boston, MA, USA; 8 Division of Endocrinology, Diabetes and Metabolism, Beth Israel Deaconess Medical Center and Harvard Medical School, Boston, MA, USA; 9 Duke Molecular Physiology Institute, Duke School of Medicine, Department of Medicine, Division of Endocrinology, Metabolism and Nutrition, Department of Pharmacology and Cancer Biology, Duke University, Durham, NC, USA; 10 Duke Molecular Physiology Institute, Duke School of Medicine, Sarah W. Stedman Nutrition and Metabolism Center, Department of Medicine, Division of Cardiology, Duke University, Durham, NC, USA; 11 Division of Metabolic Medicine, Research Center for Advanced Science and Technology, The University of Tokyo, Tokyo, Japan; 12 Division of Molecular Physiology and Metabolism, Tohoku University Graduate School of Medicine, Sendai, Japan; 13 Contributed equally; 14 Contributed equally; 15 Lead contact

**Keywords:** Brown adipose tissue, Glucose homeostasis, Inter-organ communication, Amino Acid Metabolism, Mitochondria

## Abstract

Brown adipose tissue (BAT) is best known for thermogenesis. Rodent studies demonstrated that enhanced BAT thermogenesis is tightly associated with increased energy expenditure, reduced body-weight, and improved glucose homeostasis. However, human BAT is protective against type 2 diabetes independent of body-weight. The mechanism underlying this dissociation remains unclear. Here, we report that impaired mitochondrial catabolism of branched-chain amino acids (BCAA) in BAT, by deleting mitochondrial BCAA carrier (MBC), caused systemic insulin resistance without affecting energy expenditure and body-weight. Brown adipocytes catabolized BCAA in the mitochondria as nitrogen donors for the biosynthesis of non-essential amino acids and glutathione. Impaired mitochondrial BCAA nitrogen flux in BAT resulted in increased oxidative stress, decreased hepatic insulin signaling, and decreased circulating BCAA-derived metabolites. A high-fat diet attenuated BCAA-nitrogen flux and metabolite synthesis in BAT, whereas cold-activated BAT enhanced the synthesis. The present work uncovers a metabolite-mediated pathway through which BAT controls metabolic health beyond thermogenesis.

## In Brief

Brown fat contributes to systemic glucose homeostasis by providing BCAA nitrogen-derived metabolites including glutathione to the liver, a function that’s distinct from the well-known role in thermogenesis.

## Graphical Abstract

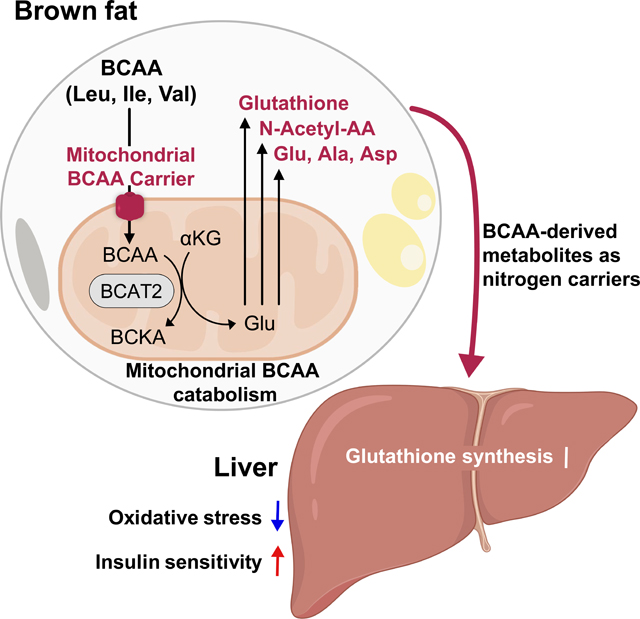

## INTRODUCTION

Exercise undoubtedly burns calories, but the metabolic benefits go far beyond increasing energy expenditure and body-weight loss. The benefits include enhanced metabolic flux and cardiac output, protection against oxidative stress, organ-cross talk through secretory factors, and more ^1,2^. Is this concept applicable to metabolic benefits associated with enhanced brown fat activity?

The most well-established function of brown and beige fat is thermogenesis by uncoupling protein 1 (UCP1) ^3^. Accordingly, the prevailing theory on BAT-associated metabolic benefit is primarily through increasing whole-body energy expenditure and the subsequent reduction in body-weight ^4^. This model is supported by numerous rodent models; however, this is not always the case in adult humans. For instance, BAT prevalence was positively associated with favorable cardiometabolic health, including lower HbA1c, lower fasting glucose, lower insulin levels, and lower cardiovascular risk, while these changes were found to be independent of resting energy expenditure or body-mass index ^5–7^. Furthermore, enhanced recruitment of human BAT by chronic cold acclimation or pharmacological activation of β3-adrenergic receptor improved whole-body insulin sensitivity, although no change in body-weight was seen after the treatment ^8,9^. The molecular basis underlying this dissociation remains less understood.

Recent studies proposed mechanisms by which BAT promotes metabolic health, including secretion of BAT-derived peptides and lipids, enhanced tissue remodeling, repression of adipose tissue inflammation, and metabolic flux of glucose, fatty acids, and BCAA ^10^. Enhanced BCAA clearance through BAT is of particular interest to us given the tight association between elevated circulating BCAA levels and type 2 diabetes in humans ^11–15^. Importantly, studies in rodents and humans demonstrated that enhanced BCAA oxidation or dietary restriction of BCAA leads to improved insulin sensitivity ^16–20^, suggesting that BCAA metabolism plays a role in the pathogenesis of insulin resistance. However, a quandary lies in the fact that BCAA is not a major carbon source to the TCA cycle in brown adipocytes even though BAT actively takes up BCAA ^21–24^. Stable isotope tracing studies showed that the contribution of BCAA carbons as a downstream substrate in the TCA cycle is far less than the respective contributions of glucose, lactate, and fatty acids in the BAT ^25–28^. Furthermore, BCAA is utilized for *de novo* lipogenesis ^29^. Accordingly, we set forth to better understand how brown adipocytes catabolize BCAA in the mitochondria other than a carbon substrate in the TCA cycle.

## RESULTS

### BCAAs are key nitrogen donors in brown adipocytes.

**Figure 1.**
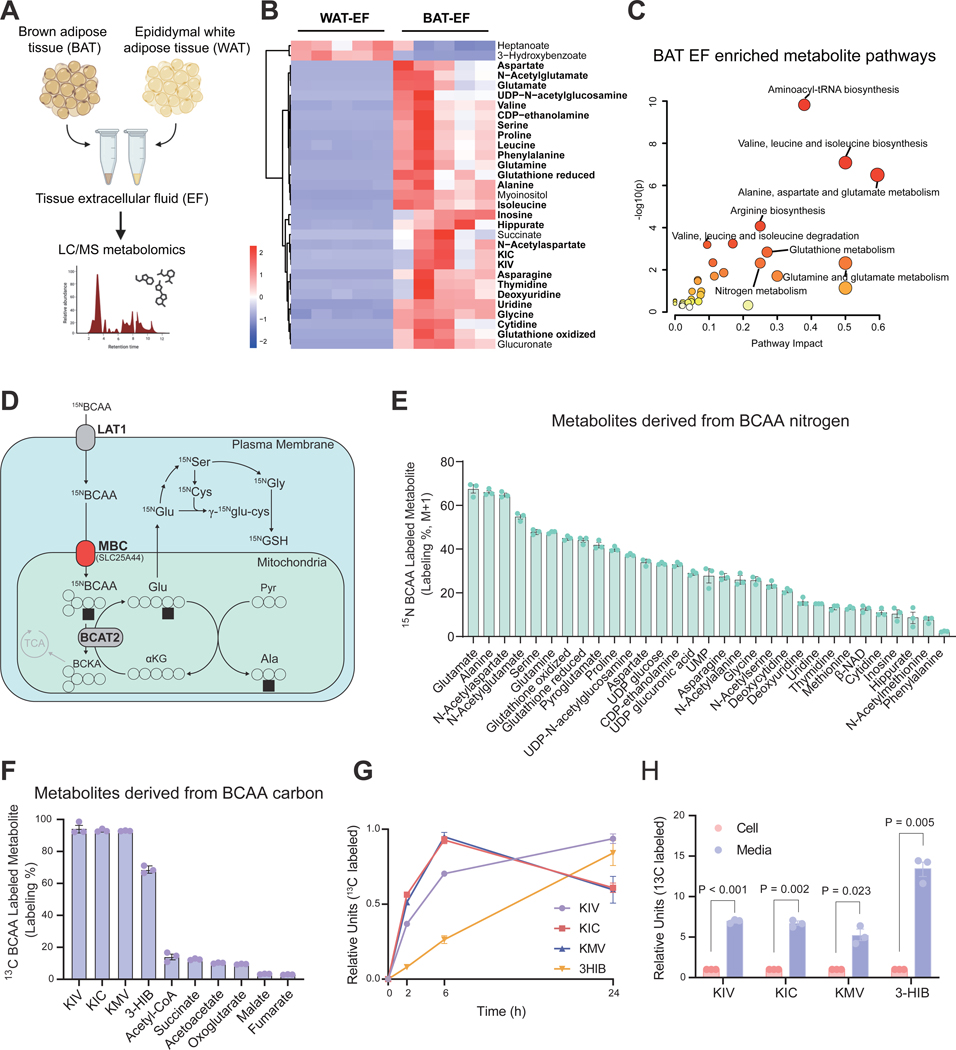
BCAAs are key nitrogen donors in brown adipocytes. **A.** Schematic of metabolomics studies in BAT vs. WAT-derived extracellular fluid (EF). **B.** Relative abundance of indicated metabolites in the EF from wild-type male mouse BAT and epidydimal WAT. Data represented as z-score heatmap for indicated metabolites. N=5 per group. **C.** The metabolite pathway analysis of BAT-EF enriched metabolites. The node color of each pathway is determined by the P-value, and the node size is based on the pathway impact factor, with the biggest indicating the highest impact. **D.** Schematic of ^15^N-BCAA tracing in brown adipocytes (nitrogen: black square, carbon: white circle). **E.** List of cellular ^15^N-metabolites derived from transamination of ^15^N-BCAA. Differentiated brown adipocytes were cultured with ^15^N-BCAA (1.6 mM each) for 24 hours. Labeling (%) represents M+1. N = 3 per metabolite. **F.** List of cellular ^13^C-metabolites derived from ^13^C-BCAA. Differentiated brown adipocytes were cultured with ^13^C-BCAA (1.6 mM each) for 2 hours. Labeling (%) represents the fraction of ^13^C labeling in all isotopomers. N = 3 per metabolite. **G.** Time course changes in indicated ^13^C-metabolite abundance in the culture media of brown adipocytes. N = 3 per group per time point. **H.** Abundance of indicated ^13^C-metabolites in the culture media and brown adipocytes. Following incubation with ^13^C-BCAAs, media was collected at 2 hours. N = 3 per group per time point.

Many BCAA-derived metabolites are secreted from cells and present in circulation ^30,31^. Hence, we first aimed to gain a holistic view of BAT-derived metabolites by performing liquid chromatography-mass spectrometry (LC-MS) metabolomics. To this end, we adapted the recently established protocol ^32^ and isolated extracellular fluids (EF) from interscapular BAT and epidydimal WAT depots of mice (Figure 1A). Metabolomics identified that α-ketoisocaproic acid (KIC), α-ketoisovaleric acid (KIV), glutamate (Glu), N-acetylglutamate (N-acetyl-Glu), N-acetylasparatate (N-acetyl-Asp), and glutathione, were significantly enriched in BAT-derived extracellular fluids relative to those from WAT (Figure 1B). We validated the observation in an independent metabolomics analysis of BAT-derived extracellular fluids (Figure S1A). The pathway analysis of BAT-EF-enriched metabolites showed significant enrichments in BCAA catabolic pathways (Figure 1C).

In highly catabolic cells including brown adipocytes, BCAAs are actively imported into the mitochondrial matrix via a mitochondrial BCAA carrier (MBC, encoded by *Slc25a44*), where they undergo deamination by BCAA transaminase 2 (BCAT2), which produces Glu and subsequently alanine (Ala) (Figure 1D). ^15^N-BCAA tracing experiments in brown adipocytes found that BCAA-derived nitrogen was utilized for the synthesis of Glu, Ala, proline (Pro), glycine (Gly), and their downstream products, including N-acetyl-Glu, N-acetyl-Asp, and reduced and oxidized forms of glutathione (Figure 1E). Although Glu and Ala can be generated *de novo* from multiple reactions, the accumulation of ^15^N-labeled Glu and Ala from BCAA occurred rapidly in the cell and was stably labeled at nearly 60%, suggesting that BCAAs are a considerable nitrogen source for the synthesis of these amino acids. This is consistent with a previous study *in vivo* that BCAA contributes to ~60% of the nitrogen needed for Ala synthesis in humans ^33^. Transamination from BCAA to these metabolites occurred rapidly and was sustained under stable-labeled conditions (Fig. S1B). The finding was consistent under several media conditions (Fig. S1C) and regardless of whether ^15^N-Val or ^15^N-Leu alone was used as a tracer (Fig. S1D).

In contrast, the results of ^13^C-BCAA tracing experiments provided a different perspective. Under the condition in which more than 90% of BCAA α-ketoacids (BCKA – KIC, KIC, KMV) were ^13^C-labeled in brown adipocytes, the labeling percentage of downstream metabolites, particularly in the TCA cycle intermediates, was lower than 10% or even less (Figure 1F). Notably, ^13^C-labeled BCKA and 3-hydroxyisobutyrate (3-HIB) were abundantly secreted from brown adipocytes (Figure 1G). The direct comparison between intracellular and secreted levels showed that the majority of the ^13^C-labeled BCKA and 3-HIB were secreted outside of brown adipocytes (Figure 1H). These results aligned with recent studies that the contribution of BCAA to TCA cycle intermediates is modest ^25–28^. Rather, BCAA is used as a considerable nitrogen donor for synthesizing non-essential amino acids and the downstream metabolites, while BCAA-derived carbons are released as BCKA and 3-HIB from brown adipocytes.

### MBC is required for the synthesis of BCAA-derived metabolites.

**Figure 2.**
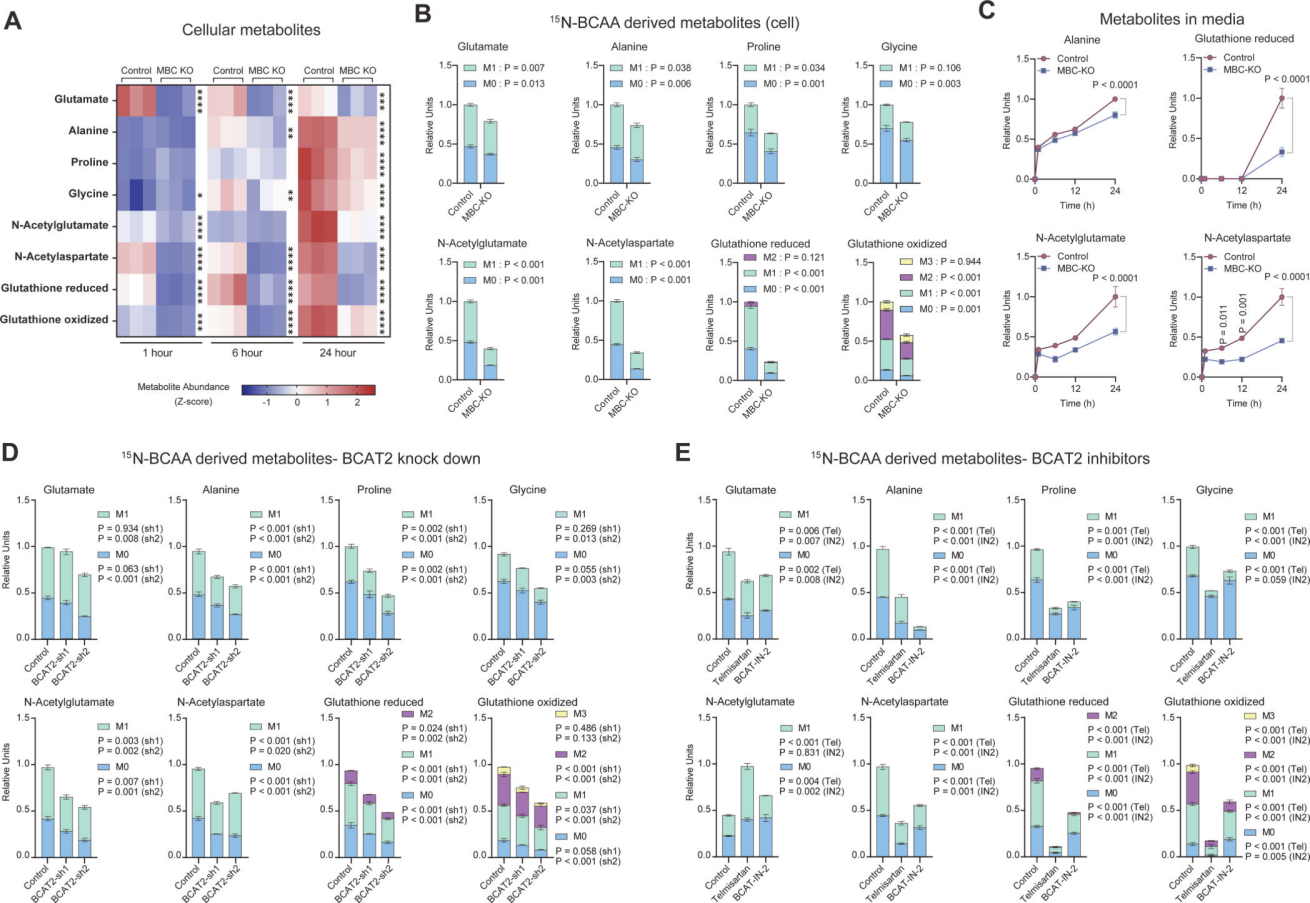
MBC is required for the synthesis of BCAA-derived metabolites. **A.** Total cellular metabolite abundance (M+0 and M+1) in control and MBC KO brown adipocytes incubated with ^15^N-BCAA at indicated time points. Data represented as z-score heatmap across 1–24 hours for each metabolite, with each cell representing quantitated value for each biological replicate. N = 3 per group per time point. Statistic for A-E: 2-way ANOVA with Šídák’s multiple comparisons test. *P<0.05; **P<0.01; ***P<0.001; ****P<0.0001. **B.** Labeled metabolites in control and MBC KO brown adipocytes cultured with ^15^N-BCAA for 24 hours. M+0, M+1, M+2, and M+3 labeled metabolites are relative to control group. N = 3 per group. **C.** Time course changes in indicated metabolite abundance (M+0 and M+1) in culture media from control or MBC KO brown adipocytes. N = 3 per group per time point. **D.** Labeled metabolites in control and shRNA-*Bcat2* brown adipocytes cultured with ^15^N-BCAA for 24 hours. M+0, M+1, M+2, and M+3 labeled metabolites are relative to control group. N = 3 per group. **E.** Labeled metabolites in brown adipocytes under control and BCAT2 inhibitors (Telmisartan and BCAT-IN-2) cultured with ^15^N-BCAA for 24 hours. N = 3 per group.

Consistent with the previous works ^24,27^, we found that MBC KO cells showed significantly less BCAA uptake in the mitochondria with resultant accumulation of BCAA within the intracellular space (Fig. S2A, B). These results reinforced the essential role of MBC for mitochondrial BCAA catabolism in brown adipocytes. Importantly, the amount of labeled nitrogen transfer from BCAA to Glu and Ala was significantly lower in MBC KO cells compared to controls (Figure 2A). Nitrogen labeling from ^15^N-BCAA to downstream non-essential amino acids and metabolites, including Pro, Gly, N-acetyl-Glu, N-acetyl-Asp, as well as reduced and oxidized forms of glutathione, were reduced in MBC KO cells (Figure 2B). Similarly, when ^15^N-Val was individually used as a tracer, nitrogen transfer from Val to these metabolites occurred in an MBC-dependent manner (Figure S2C). Since many *de novo* synthesized BCAA-nitrogen derived metabolites are enriched in the extracellular fluid of BAT (see Fig. 1B), we then measured ^15^N-labeled metabolites in the cell culture media of differentiated brown adipocytes. We found that nearly all the BCAA-derived metabolites, including Ala, N-acetyl-Glu, N-acetyl-Asp, and glutathione, were abundantly secreted from brown adipocytes, while the amounts were significantly lower in MBC KO secreted milieu relative to those from control cells (Figure 2C, Figure S2D).

Next, we determined if BCAT2 inhibition recapitulated the loss of MBC in the synthesis of BCAA-nitrogen-derived metabolites. To this end, we employed two complementary approaches; short hairpin RNA (shRNA)-mediated depletion of BCAT2 and pharmacological inhibition of BCAT2. Firstly, we developed two independent shRNAs targeting BCAT2 in brown adipocytes (Figure S2E). Subsequently, we performed ^15^N-BCAA tracing in control and BCAT2-depleted brown adipocytes under the same condition as MBC KO cells. We found that depletion of BCAT2 resulted in a significant reduction in the levels of ^15^N-labelled Glu, Ala, Pro, Gly, N-acetyl-Glu, N-acetyl-Asp, and glutathione (Figure 2D). ^15^N-labelled Glu levels in BCAT2-shRNA#1 cells were significantly lower than control cells at 2 hours, but not at 24 hours (Figure S2F). Next, we performed ^15^N-BCAA tracing in brown adipocytes treated with two small compounds, telmisartan and BCAT-IN-2 ^34,35^. Consistent with the results in shRNA knockdown, BCAT2 inhibition by telmisartan significantly reduced the levels of ^15^N-BCAA-derived metabolites (Figure 2E). The inhibitory effect of telmisartan on the synthesis of ^15^N-metabolites largely recapitulated the metabolite changes following shRNA-mediated knockdown of BCAT2 with the exception of N-acetyl-Glu. We found a similar trend when BCAT2 was inhibited by BCAT-IN-2.

Nonetheless, it is worth pointing out the differences between MBC loss and BCAT inhibition. BCAT2 inhibition leads to BCAA accumulation, whereas the blockade of MBC resulted in reduced mitochondrial BCAA contents and the accumulation in the cytosolic compartment (Figure S2B). Besides, MBC KO cells contained detectable levels of BCKA, albeit to a significantly lesser extent (Figure S2G), which might arise from the cytosolic deamination of BCAA by BCAT1 that is expressed in brown fat despite low levels ^36^. Of note, supplementation of KIV restored oxygen consumption rate (OCR) in MBC KO adipocytes (Fig. S2H). These results suggest that BCKAs are imported into the mitochondrial compartment of MBC KO cells. Additionally, MBC KO cells showed higher levels of glycolytic intermediates than control cells, suggesting that loss of MBC shifted cellular metabolism towards glycolysis (Fig. S2I).

### Mechanisms of mitochondrial BCAA catabolism via MBC.

**Figure 3.**
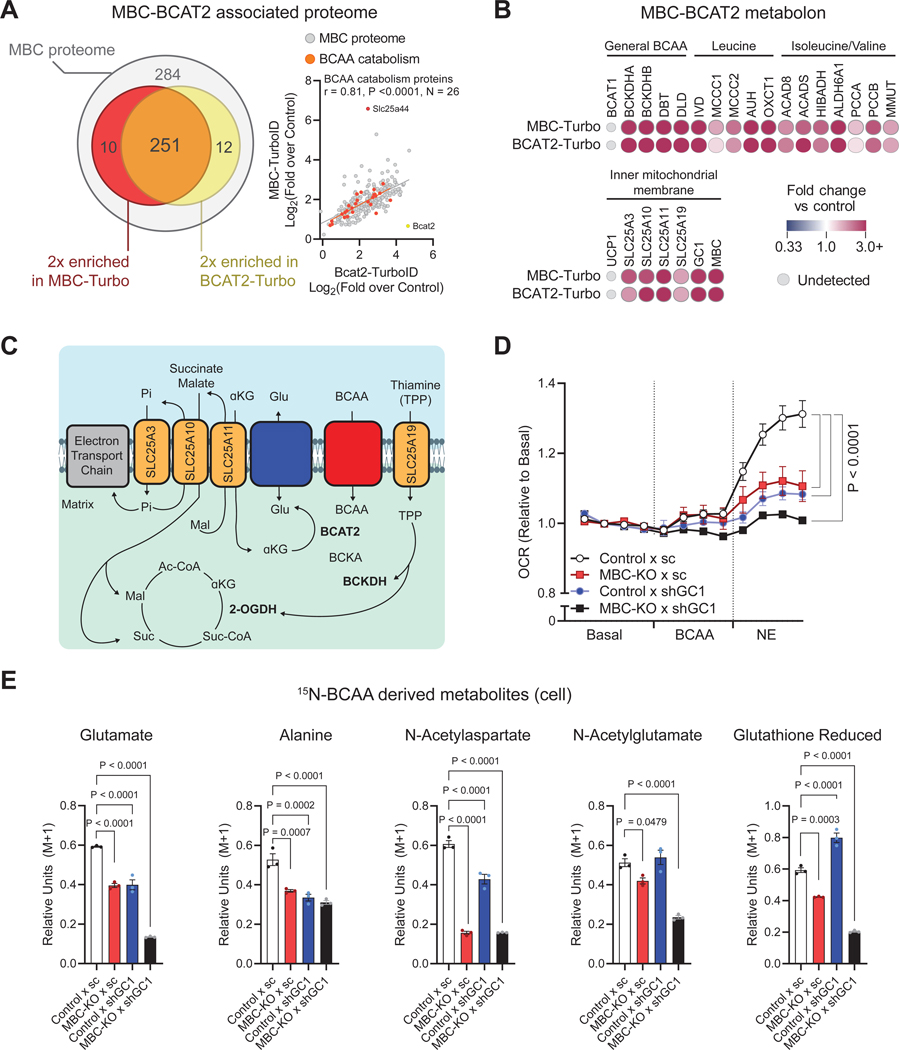
Mechanisms of mitochondrial BCAA catabolism via MBC. **A.** Left: Venn Diagram of the MBC-proteome comparing enriched proteins (≥ 2-fold) from MBC-TurboID with BCAT2-TurboID proximity labeling. Right: MBC proteome correlated between MBC-TurboID and BCAT2-TurboID fold change compared to control. **B.** Mitochondrial BCAA catabolic protein identified in BCAT2-TurboID and MBC-TurboID brown adipocytes. Data are represented as fold change over empty-vector controls. Undetected proteins are shown in grey. **C.** Inner mitochondrial membrane proteins identified in MBC-Flag-pulldown and MBC-TurboID experiments. **D.** OCR of differentiated brown adipocytes in response to BCAA and norepinephrine at indicated time points. N = 5 per group. Statistic: 2-way ANOVA with Dunnett’s multiple comparisons test. **E.** ^15^N-BCAA labeled (M+1) metabolites detected in indicate brown adipocytes incubated with ^15^N-BCAA (1.6 mM) for 24 hours. N = 3 per group. Statistic: one-way ANOVA with Dunnett’s multiple comparisons test.

To better understand the mechanism of mitochondrial BCAA catabolism through MBC, we next performed unbiased interactome analyses in differentiated brown adipocytes stably expressing MBC tagged with FLAG or Turbo-ID proximity labeling tag. MBC-associated proteins were detected by FLAG-immunoprecipitation (IP) and biotinylated peptides using Turbo-ID, followed by tandem mass tag (TMT) based quantitative proteomics. The data were then curated by using the reference protein list in the MitoCarta 3.0 dataset ^37^. In total, we identified 284 mitochondrial proteins that are associated with MBC between the two methods (Fig. S3A, Table S1). Pathway analysis found that the BCAA catabolic process is one of the most significantly enriched metabolic pathways in the MBC-interactome (Fig. S3B, C). This finding is intriguing given the notion of a “BCAA metabolon” wherein BCAA catabolic enzymes, including BCAT2, form a supramolecular complex within the mitochondria for efficient BCAA catabolism ^38–40^. Accordingly, we next performed Turbo-ID proximity labeling of BCAT2 in brown adipocytes and overlayed the dataset with the MBC-associated proteome. Strikingly, nearly 95% (251/263 proteins) of BCAT2-associated proteins were found to be enriched in the MBC-associated proteome (Figure 3A). The MBC-BCAT2 proteome included mitochondrial BCAA catabolic enzymes, including the BCKDH complex, IVD, ACAD8, and ACADS (Figure 3B). Note that the enrichment of the BCAA catabolic pathway was not due to protein abundance because UCP1, one of the most abundant mitochondrial proteins in brown fat, was not detected in either proteomics.

In the subsequent analysis of this proteomic approach, we also identified several mitochondrial inner-membrane proteins, including the thiamine pyrophosphate carrier (TPP, SLC25A19), glutamate carrier (GC1, encoded by *Slc25a22*), oxoglutarate carrier (SLC25A11), dicarboxylic acid carrier (SLC25A10), and phosphate carrier (SLC25A3), as being in proximity to the MBC (Figure 3B, C). The result provided insights into the mechanism of MBC action as these SLC25A carriers are essential for mitochondrial BCAA catabolism. For instance, the first step of mitochondrial BCAA metabolism requires α-ketoglutarate (α-KG) as a substrate to form glutamate; these metabolites are transported by SLC25A11 and GC1, respectively. The second and rate-limiting step of mitochondrial BCAA metabolism requires thiamine as a co-factor, which is regulated by SLC25A19. Furthermore, the presence of SLC25A11, SLC25A10, and SLC25A3 in the MBC metabolon provides a synchronous balance of anaplerotic and cataplerotic intermediates for the TCA cycle.

The above results suggest the possibility that GC1 supports mitochondrial BCAA catabolism by transporting mitochondrial Glu derived from transamination of BCAA. To test this hypothesis, we next utilized shRNA to generate brown adipocytes lacking GC1 and/or MBC (Fig. S3D). Note that MBC loss did not decrease the expression of BCAA catabolic enzymes (e.g., BCAT2) and GC1 (Fig. S3E); hence, metabolic changes in MBC KO cells would not be related to down-regulated BCAA enzymes or GC1-mediated Glu transport. In these cells, we measured OCR in response to BCAA supplementation and norepinephrine stimulation. We found that depletion of MBC or GC1 did not impair the basal OCR prior to BCAA supplementation; however, either a knockdown of GC1 or knockout of MBC was sufficient to blunt cellular respiration in response to BCAA supplementation and norepinephrine stimulation. When both GC1 and MBC were depleted, BCAA-induced cellular respiration was further blunted (Figure 3D). This functional interaction between MBC and GC1 was also found in the synthesis of BCAA-derived metabolites. A knockout of MBC was sufficient to reduce metabolite pools and ^15^N-BCAA-labeling into Glu, Ala, N-acetyl-Asp, N-acetyl-Glu, and glutathione. When both MBC and GC1 were depleted, the metabolite pool and ^15^N labeling of Glu, Ala, N-acetyl-Glu, and glutathione were further reduced (Figure 3E, Fig. S3F).

### Impaired BCAA flux in BAT causes insulin resistance independent of energy expenditure.

**Figure 4.**
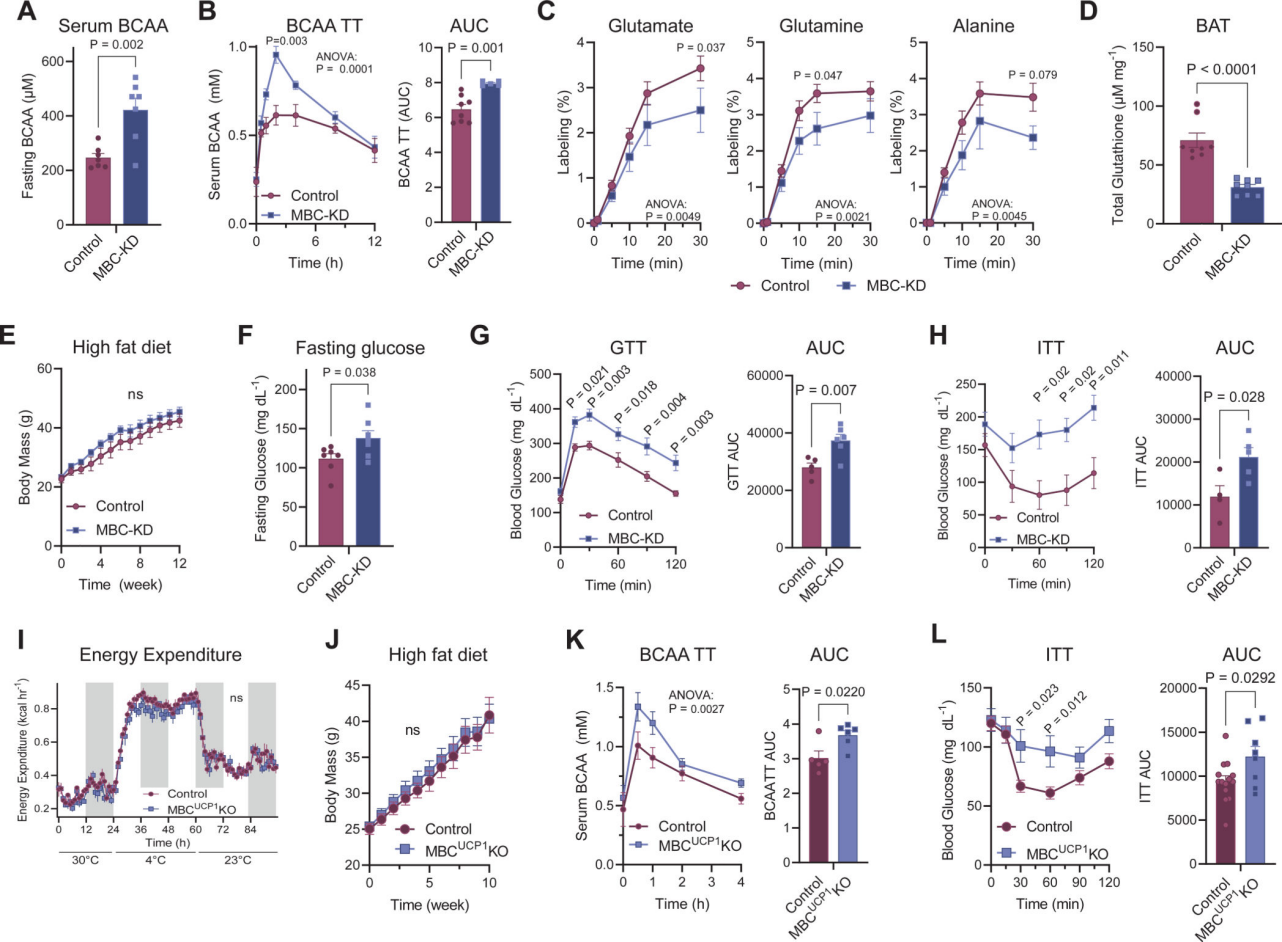
Impaired BCAA flux in BAT causes insulin resistance independent of energy expenditure. **A.** Serum BCAA from 4-hour fasted control and MBC-KD male mice fed a high-fat diet. N = 7 per group. **B.** BCAA tolerance test and area under the curve (AUC) of control and MBC-KD male mice. Changes in blood BCAA levels were measured in fasted mice in response to an oral bolus BCAA challenge (75 mg kg^−1^). N = 8 control, 5 MBC-KD. **C.** ^15^N-BCAA derived Glu, Gln, and Ala in the serum of MBC-KD and control male mice. **D.** Total glutathione (reduced and oxidized) amount in BAT of control and MBC-KD male mice. N = 8 per group. **E.** Body mass of control and MBC-KD male mice on a high-fat diet. N = 7 control and 10 for MBC-KD. **F.** Serum glucose from 4-hour fasted control and MBC-KD male mice fed a high-fat diet. N = 7 per group. **G.** Glucose tolerance test and AUC of high-fat diet fed control and MBC-KD male mice. Mice were fasted for 4 hours prior to collecting baseline blood glucose measurement and subsequent intraperitoneal injection of glucose (1 g kg^−1^). N = 5 control mice and 6 for MBC-KD mice. **H.** Insulin tolerance test and AUC of mice in (G). Mice were fasted for 4 hours prior to intraperitoneal injection of insulin (1 U kg^−1^). N = 4 control mice and 5 for MBC-KD mice. **I.** Energy expenditure of control male mice and male mice with brown fat knockout of MBC (MBC^UCP1^ KO) at indicated temperature. N = 4 control mice and 3 for MBC^UCP1^ KO mice. **J.** Body mass of control and MBC^UCP1^ KO male mice on a high-fat diet. N = 8 per group. **K.** BCAA tolerance test and AUC of control and MBC^UCP1^ KO male mice. Changes in blood BCAA levels were measured in fasted mice in response to an oral bolus BCAA challenge (75 g kg^−1^). N = 5 control mice and 6 for MBC^UCP1^ KO mice. **L.** Insulin tolerance test and AUC of control and MBC^UCP1^ KO male mice. Mice were fasted for 4 hours prior to intraperitoneal injection of insulin (1 U kg^−1^). N = 14 control mice and 8 for MBC^UCP1^ KO mice.

We next analyzed the metabolic phenotype of mice lacking MBC (MBC-KD) by employing CRISPRi technology (Fig. S4A). In circulation, MBC-KD mice exhibited significantly higher levels of serum BCAA levels than controls under a fasted condition (Figure 4A). This elevation in serum BCAA levels was due to impaired BCAA clearance because MBC-KD mice showed impaired BCAA tolerance in response to an oral BCAA challenge (Figure 4B). We then examined if MBC would be required for the synthesis of BCAA-nitrogen derived metabolites *in vivo* by administering ^15^N-BCAA to mice via *i.p.* injection. Note that increased levels of ^15^N-BCAA proportionally resulted in elevated ^15^N-BCAA derived Glu, Gln, and Ala in circulation (Fig. S4B, C), *a.k.a.* mass-action ^41^. Consistent with the tracing studies in cultured cells, we found significantly lower levels of ^15^N-metabolites in the serum of MBC-KD mice compared to in control mice following ^15^N-BCAA injection *in vivo* (Figure 4C). Furthermore, the BAT of MBC-KD mice contained significantly lower levels of glutathione compared to controls (Figure 4D).

When male mice were on a high-fat diet, we did not observe any difference in body-weight or tissue masses (Figure 4E, Fig. S4D). While a previous paper showed that BCAT2 loss leads to compensatory activation of beige fat biogenesis in the inguinal WAT ^35^, we found no such changes in MBC-KD mice (Fig. S4E). Nonetheless, we did observe a significant increase in blood glucose levels under a fasted condition (Figure 4F). No difference was found in circulating blood triglycerides (Fig. S4F). However, when challenged with a glucose or insulin bolus, MBC-KD mice demonstrated glucose and insulin intolerance compared to littermate controls (Figure 4G, H). On the other hand, we found no differences in fasting- and glucose-stimulated insulin levels between the genotypes (Fig. S4G). These metabolic phenotypes were consistent in female mice, including impaired glucose tolerance (Fig. S4H, I).

To determine the role of MBC specific to BAT, we next developed *Slc25a44*^flox/flox^ mice and subsequently crossed them with *Ucp1*-Cre to generate BAT-specific knockout mice (*Ucp1*-Cre; *Slc25a44*^flox/flox^, herein MBC^UCP1^ KO mice) (Fig. S4J). Then we performed indirect calorimetry on MBC^UCP1^ KO mice and littermate control mice (*Slc25a44*^flox/flox^) and examined whole-body energy expenditure at thermoneutral (30°C), cold (4°C), and ambient (23°C) temperatures. We found no significant difference in energy expenditure between the two groups, even though there was a modest but insignificant trend of lower energy expenditure in MBC^UCP1^ KO mice relative to controls at 4°C (Figure 4I, Fig. S4K). Additionally, there was no difference in cold tolerance between the groups (Fig. S4L). This is in alignment with the results that BCAA is not the major carbon fuel for oxidation in BAT, and hence, BAT-specific MBC loss can be compensated by glucose and fatty acids *in vivo* under physiological conditions. We also found no differences in food intake or locomotor activity between control and MBC^UCP1^ KO mice (Fig. S4M, N). Accordingly, there was no difference in body mass gain and body composition between the two genotypes (Figure 4J, Fig. S4O). However, MBC^UCP1^ KO exhibited significantly lower BCAA clearance than control mice in response to a BCAA bolus (Figure 4K). Importantly, MBC^UCP1^ KO showed impaired insulin tolerance relative to littermate controls (Figure 4L). Together, these results suggest that an impaired BCAA flux and subsequent decline in the synthesis of BCAA-derived metabolites in BAT sufficiently attenuates insulin tolerance without affecting whole-body energy expenditure and body weight.

### Impaired BCAA flux in BAT induces oxidative stress.

**Figure 5.**
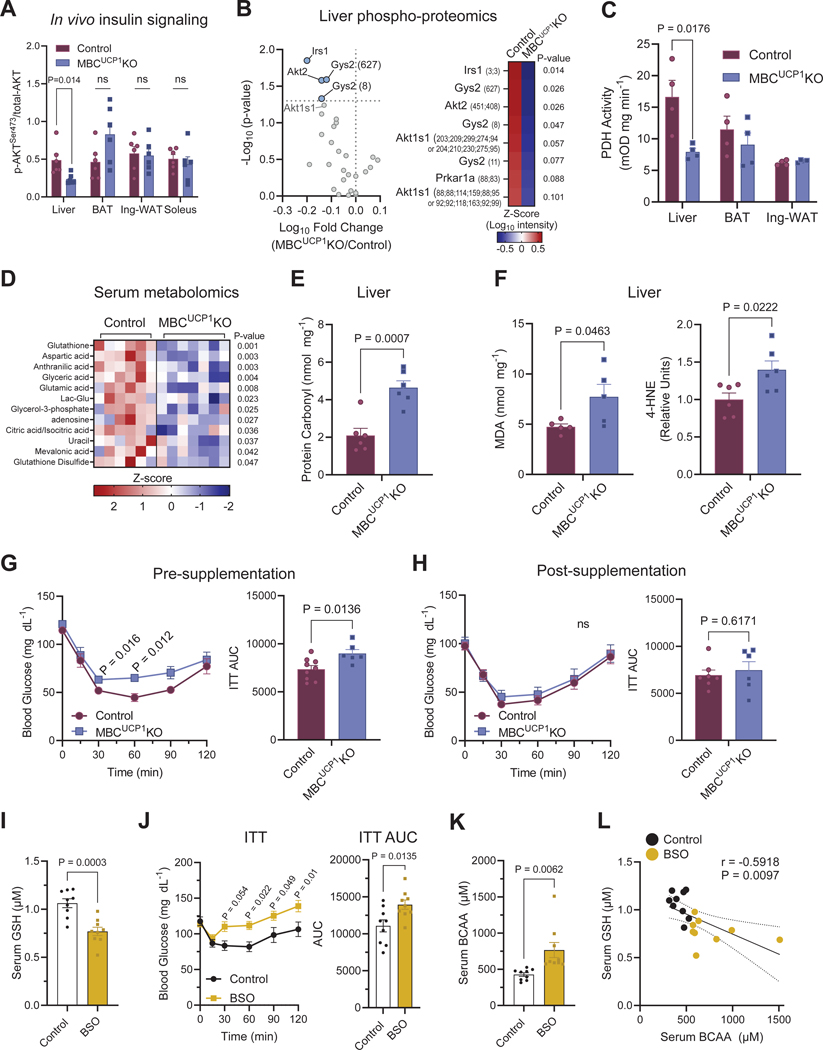
Reduced glutathione mediates insulin resistance. **A.** *In vivo* insulin stimulation of AKT^Ser473^ phosphorylation in indicated tissues (phospho-AKT^Ser473^ over total-AKT). Male mice were fasted 4 hours prior to insulin injection. N = 6 for both groups. **B.** Phospho-proteomic analysis of *in vivo* insulin-stimulated liver tissues in (A). Left: Scattered plot of the analysis with indicated phosphor-proteins. Right: Heat map of averaged phosphor-proteins. N = 6 from control and N = 5 from KO liver, male mice. **C.** PDH activity in response to insulin in (A). N = 4 per group per each tissue. **D.** Metabolomics in serum from high-fat diet fed control and MBC^UCP1^ KO male mice. N = 6 for control and 7 for MBC^UCP1^ KO. **E.** Oxidative stress marker protein carbonyl content in the liver from control and MBC^UCP1^ KO male mice on a high-fat diet. N = 6 per group. **F.** Abundance of lipid oxidative stress marker malondialdehyde (MDA) content (left) and 4-hydroxylnonenal (4-HNE) content (right) in liver tissue. N = 5 per group for MDA content, N = 6 per group for 4-HNE quantification. **G.** Insulin tolerance test and AUC of high-fat diet fed control and MBC^UCP1^ KO male mice prior to glutathione supplementation. Mice were fasted for 4 hours prior to intraperitoneal injection of insulin (1 U kg^−1^). N = 9 control and 6 for MBC^UCP^ KO mice. **H.** Insulin tolerance test and AUC of male mice in (G) following 10 days of glutathione supplementation (2 g kg^−1^). Mice received glutathione supplementation 18 hours prior to insulin tolerance test to limit acute glutathione response. N = 7 control mice and 6 for MBC^UCP^ KO mice. **I.** Serum GSH levels in wild-type male mice following BSO treatment. N = 9 per group. **J.** Insulin tolerance test (0.5 U kg^−1^) in wild-type male mice treated with BSO for 20 days. N = 9 per group. **K.** Serum BCAA levels in (J). **L.** Correlation between serum BCAA and serum GSH levels in (J).

To determine which organs were responsible for systemic insulin resistance in MBC^UCP1^ KO mice, we assessed AKT phosphorylation as a marker of insulin signaling in peripheral metabolic organs collected from MBC^UCP1^ KO mice and controls that received bolus insulin on a fasted condition. We found that MBC^UCP1^ KO mice had blunted insulin-induced phosphorylation of AKT^Ser473^ in the liver, but not in BAT, inguinal WAT, or skeletal muscle (Figure 5A, Fig. S5A). The phospho-proteomics analyses found that phosphorylation of Irs1, Akt2, and Gys2 (the liver isoform of glycogen synthase) was significantly lower in MBC^UCP1^ KO mice compared to controls (Figure 5B). Additionally, we observed trends of reduced phosphorylation of known phosphorylated sites of Akt signaling, including Akt1s1. Furthermore, hepatic PDH activity was significantly reduced in MBC^UCP1^ KO mice compared to controls, while PDH activities in BAT and inguinal WAT were unchanged (Figure 5C). These observations point toward BCAA catabolism in BAT having an impact on liver function.

How does impaired BCAA catabolism in BAT lead to hepatic insulin resistance without affecting energy expenditure? Since we did not find any difference in the hepatic contents of triglyceride, diacylglycerol, ceramide, and glucosylceramide between the genotypes (Fig. S5B, C), we next performed serum metabolomics to determine any change in circulating metabolites. The serum metabolomics identified several circulating metabolites that were significantly reduced in MBC^UCP1^ KO mice relative to controls, including glutamic acid, aspartic acid, glutathione, and glutathione disulfide (Figure 5D). Notably, many of these metabolites were identified as BCAA-nitrogen-derived metabolites via MBC in brown adipocytes. Moreover, the livers of MBC^UCP1^ KO mice exhibited significantly elevated levels of oxidative stress markers, including protein carbonyl content (Figure 5E) and lipid peroxidation markers malondialdehyde (MDA) and 4-hydroxy-2-nonenal (4-HNE) (Figure 5F, Fig. S5D).

These results led to the hypothesis that BCAA catabolism in BAT impacts systemic redox balance. To test this, we supplemented glutathione to control and MBC^UCP1^ KO mice to restore the observed reduction in glutathione levels in MBC^UCP1^ KO mice. In an independent cohort, we validated that MBC^UCP1^ KO mice exhibited impaired insulin tolerance prior to glutathione supplementation (Figure 5G). After ten days of supplementation, serum levels and liver contents of total glutathione became indistinguishable between control and MBC^UCP1^ KO mice (Fig. S5E, F). Importantly, insulin intolerance in MBC^UCP1^ KO mice was completely restored following glutathione supplementation (Figure 5H). Of note, glutathione supplementation to diet-induced obese wild-type mice improved systemic insulin tolerance without affecting body weight (Fig. S5G–I). Regardless, glutathione supplementation was potent enough to restore the insulin tolerance of MBC^UCP1^ KO mice to a level comparable to glutathione-supplemented control mice. Furthermore, insulin-induced AKT^Ser473^ phosphorylation in the liver of MBC^UCP1^ KO mice was fully restored to the levels seen in control mice following glutathione treatment (Fig. S5J). Conversely, we aimed to block glutathione synthesis by pharmacologically inhibiting glutamate-cysteine ligase using buthionine sulphoximine (BSO) *in vivo*. As expected, BSO treatment for 5 weeks significantly reduced serum glutathione levels (Figure 5I). BSO treatment in mice also attenuated insulin tolerance even though BSO-treated mice lost body weight relative to vehicle-treated mice (Figure 5J, Fig. S5K). It is notable that BSO treatment resulted in elevated BCAA levels in circulation (Figure 5K). Furthermore, we found a significant inverse correlation between serum BCAA and glutathione levels (Figure 5L). Together, these results suggest that impaired mitochondrial BCAA flux and subsequent reduction in the synthesis of BCAA-derived metabolites in BAT leads to elevated oxidative stress and reduced insulin signaling in the liver.

### BCAA catabolism in BAT is coupled with glutathione synthesis.

**Figure 6.**
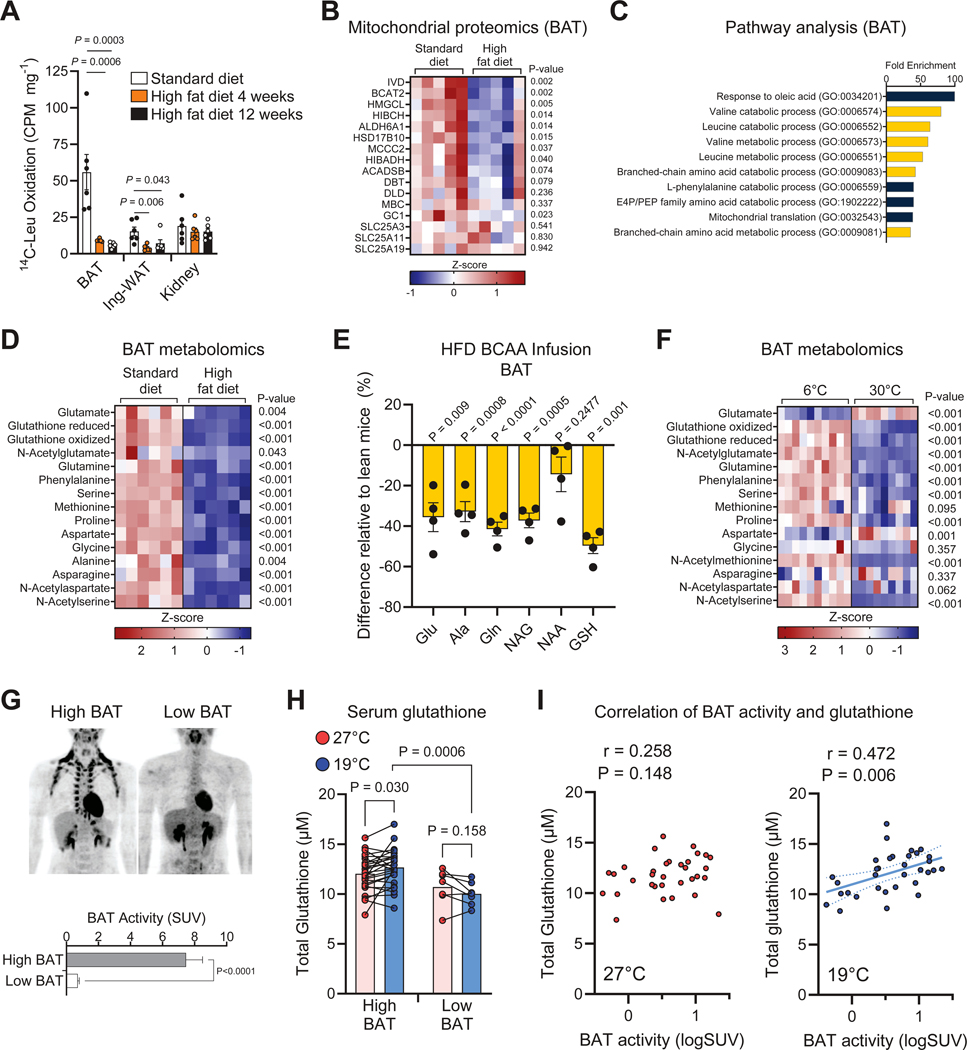
BCAA catabolism in BAT is coupled with glutathione synthesis. **A.** *Ex vivo* ^14^C-Leu oxidation in wild-type male mice fed a standard diet or a high-fat diet for 4 and 12 weeks. N = 6 per group. **B.** Indicated protein abundance in BAT mitochondria of wild-type male mice fed a standard diet or high-fat diet for 8 weeks. Isolated mitochondria were subjected to quantitative proteomics. N = 5 per group. **C.** Gene ontology of biological processes from all the down-regulated BAT mitochondrial proteins in (B). **D.** BCAA-linked metabolites in the BAT of wild-type male mice fed a standard diet or a high-fat diet for 12 weeks. N = 6 per group. **E.** Changes in indicated ^15^N-labeled metabolites in the BAT of male mice stably infused with ^15^N-BCAA for 12 hours. N = 4 per group. **F.** BCAA-linked metabolites in the BAT of wild-type male mice acclimated to 6°C or 30°C for two weeks. N = 10 for mice housed at 6°C and 9 for mice housed at 30°C. **G.** Representative ^18^FDG-PET of high BAT (SUV ≥ 2.0) and low BAT (SUV< 2.0) groups. Quantification of BAT activity (SUV) is shown below. N = 26 for high BAT and 7 for low BAT. **H.** Total glutathione in the serum of subjects at 27°C and after 2 hours of exposure to 19°C in (G). Statistic between 27°C and 19°C is Wilcoxon matched-pairs signed rank test with multiple comparisons corrected by two-stage step up (Benjamini, Krieger, and Yekutieli) method. Statistic comparing high BAT 19°C to low BAT 19°C is Mann Whitney test. **I.** Correlation between BAT activity (log SUV) and circulating glutathione levels in all the subjects examined in the study (N=33) following cold exposure at 19°C and at 27°C by Spearman correlation two-tailed test.

How is the synthesis of BCAA-derived metabolites regulated in physiology and disease? To address this question, we first determined the extent to which diet-induced obesity impacts BCAA catabolism in metabolic organs, including iBAT, inguinal WAT, kidney, heart, and skeletal muscle. As expected, mice on short-term (4 weeks) or long-term (12 weeks) high-fat diets exhibited greater body mass and higher blood glucose levels than mice on a regular diet (Fig. S6A, B). We found that BCAA-oxidation in the iBAT was reduced compared to control mice within four weeks of a high-fat diet and thereafter (Figure 6A). A reduction in BCAA-oxidation also occurred in inguinal-WAT following short- and long-term high-fat diet-fed mice, although the degree of reduction was less compared to iBAT. In contrast, no major change was found in the kidney, liver, skeletal muscle, and heart following a high-fat diet (Fig. S6C).

To determine how obesity rapidly impairs BCAA catabolism, we next purified mitochondria from the iBAT of mice fed a standard diet or 8 weeks of high-fat diet and subsequently performed TMT-based quantitative mitochondrial proteomics. Unbiased proteomic analyses identified numerous mitochondrial proteins involved in BCAA catabolism, including BCAT2, IVD, and GC1 were significantly reduced in the BAT of obese mice than those of controls (Figure 6B). Gene ontology analysis revealed that BCAA catabolism was one of the most representative pathways that were down-regulated in diet-induced obesity (Figure 6C). The reduction in BCAA catabolic proteins was not due to a general decline in mitochondrial abundance or activity because many TCA cycle enzymes in the mitochondria were unchanged between obese vs. lean mice (Fig. S6D). Moreover, the obesity-associated decline in BCAA catabolism was accompanied by reduced levels of Glu, N-acetyl-AAs, Pro, Ala, and glutathione in BAT (Figure 6D).

To determine if obesity-associated decline in these BAT metabolites was attributed to reduced *de novo* synthesis from BCAA-derived nitrogen, we next stably infused ^15^N-BCAA into catheterized mice on a high-fat diet for 8 weeks (obese) or on a regular diet (lean). Following the previous study ^23^, we set the infusion rate to reach approximately 30% labeling of ^15^N-BCAA in circulation. Consistent with the ^13^C-BCAA tracing study by Neinast et al. ^23^, we found that obese mice exhibited a significantly lower disposal rate (Rd, nmol min^−1^ g^−1^) of ^15^N-BCAA (Fig. S6E). Importantly, the levels of ^15^N-BCAA-derived metabolites in the BAT, including ^15^N-Glu, Ala, N-Acetyl-Glu, and glutathione, were significantly lower in obese mice than in lean mice (Figure 6E). On the other hand, no major change was seen in the pancreas, the organ that actively utilizes BCAA for protein synthesis (Fig. S6F).

Because cold acclimation is known to activate BCAA uptake in brown fat ^22,24^, we next examined how temperature impacts the synthesis of BCAA-derived metabolites in BAT. Metabolomics analyses found that many of the above-mentioned BCAA-derived metabolites, such as N-acetyl-AAs and reduced and oxidized glutathione, were significantly enriched in cold-acclimated BAT relative to warm-acclimated BAT (Figure 6F). It is notable that obesity-associated changes in the BAT metabolite profile highly resembled those that occurred during acclimation to thermoneutrality at 30°C. In the circulation, we also observed similar increases in serum BCAA-derived nitrogen metabolites, such as N-acetyl-Glu and reduced glutathione (Fig. S6G).

Lastly, we examined whether cold activation of BAT in adult humans was associated with changes in circulating glutathione levels. To this end, we collected sera from healthy adult males (averaged age 23.2 years, n = 33) at 27°C and after 2-hour mild-cold exposure of 19°C (Table S2). We used positron emission tomography-computed tomography with ^18^F-fluorodeoxyglucose (^18^FDG-PET) to determine the BAT activity of the subjects. The high-BAT group had BAT activity levels of SUV ≥ 2.0 and the low-BAT group had BAT activity of SUV < 2.0 (Figure 6G). The subjects are healthy young subjects; hence, there was no difference in body weight, BMI, and fat mass between the two groups. Following cold exposure, we found that circulating levels of total glutathione in the high-BAT group were significantly higher than those in the low-BAT group (Figure 6H). Cold exposure led to a modest but significant increase in circulating glutathione levels in the high-BAT group, whereas this change was not seen in the low-BAT group. Importantly, there was a significant positive correlation between circulating glutathione levels and BAT activity (SUV) following cold exposure, while such a correlation was not seen at 27°C (Figure 6I). Together, these data suggest that enhanced BAT activity following cold exposure is coupled with increased synthesis of BCAA nitrogen-derived metabolites, including glutathione, whereas diet-induced obesity impairs BCAA catabolism and the synthesis of these metabolites in BAT.

## DISCUSSION

The present study shed light on two important quandaries in the field. Firstly, the dissociation between BAT-associated metabolic benefits and changes in body-weight is evident in humans, although studies in rodents have repeatedly demonstrated tight relationships between active brown/beige fat thermogenesis, enhanced energy expenditure, body-weight loss, and improved glucose homeostasis. Certainly, some of this can be attributed to the species difference in body mass/surface ratio and the demand for BAT-mediated non-shivering thermogenesis ^42^. However, our study showed in mice that a defect in mitochondrial BCAA catabolism specific to BAT sufficiently causes an impairment in insulin signaling without affecting whole-body energy expenditure and body-weight. Note that the roles of BAT-derived secretory peptides/lipids, *a.k.a.* batokines, on energy homeostasis have become widely appreciated; however, currently known batokines are reported to control thermogenesis and energy expenditure ^43^. The model proposed in this study is distinct from, but not contradictory to, the batokine model in that BAT contributes to the pool size of BCAA-derived metabolites in cells, tissues, and circulation, which impact glucose homeostasis without influencing overall energy expenditure. The second quandary pertains to the incremental contribution of BCAA to the TCA cycle as a carbon source, despite BAT actively taking up BCAA ^25–28^. This study shows that BCAA serves as a key nitrogen source in BAT for the synthesis of non-essential amino acids and their downstream products, including Glu, Ala, Asp, N-acetyl-AAs, and glutathione, many of which are secreted outside of brown adipocytes. Besides, BCAA-derived carbons are primarily secreted from brown adipocytes in the forms of BCKA and 3-HIB, as reported by the previous study in muscle cells ^31^, while a small fraction of BCAA-derived carbons enter the TCA cycle. It has also been shown that BCAA-derived carbons are utilized for the synthesis of mmBCFA, and Leu/Ile contribute to the acetyl-CoA pool, both of which are critical for *de novo* lipogenesis in brown fat ^29,44,45^. These results suggest that active BCAA uptake to BAT is critical for metabolite synthesis, rather than serving as carbon fuel for thermogenesis.

The present study, along with a recent publication ^46^, suggests that BAT and skeletal muscle influence systemic BCAA flux. The etiology is difficult to define; however, we speculate the following. Firstly, the liver lacks the BCAT deamination enzyme, and therefore, it depends on the supply of BCKA from other metabolic organs for oxidation, gluconeogenesis, and ketogenesis ^47^. On the other hand, BAT, which expresses BCAT2 and SLC25A44 at the highest level in mammalian tissues, may serve as a metabolic organ that supplies BCKA and nitrogen carriers to the liver. In this context, examples of inter-organ metabolite communication between skeletal muscle and the liver include the Cori cycle (lactate-glucose) and the Cahill (glucose-alanine) cycle ^48^. Second, Leu activates the mTOR complex I (mTORC1) via Sestrin1/2 and regulates cellular nutritional states ^49,50^. Of note, we found that BCAA catabolism in BAT is rapidly and selectively downregulated in an obese state, where systemic BCAA flux is reduced. It is conceivable that obesity-associated impairment of BCAA catabolism in BAT and subsequent changes in systemic BCAA flux serve as a nutrient signal to the liver to modulate mTORC1 and downstream signaling pathways.

In the context of exercise, weight-loss is not the primary metabolic outcome in the long term. Besides acutely elevating energy expenditure, activation of skeletal muscle following exercise enhances metabolite flux and stimulates the secretion of metabolites and myokines that affect the central and peripheral organs, including adipose tissues ^1,2^. Activation of BAT may provide similar corollaries of cardiometabolic benefits outside of stimulating energy expenditure. Intriguingly, winter swimmers, who regularly practice short-term cold exposures during winter, exhibit better glucose tolerance and blunted increases in blood pressure following cold exposure relative to non-cold swimmer controls, even though their body-weight and resting energy expenditure under a thermal comfort state are equivalent to controls ^51,52^. In addition, winter-swimmers have been found to exhibit higher levels of basal total and reduced glutathione levels compared to controls, which may confer cardiovascular benefits ^53,54^. Conversely, the Framingham Heart Study identified elevated oxidative stress markers and circulating BCAA levels as early predictors of insulin resistance ^13,14,55,56^. Taken together, the present work calls for new research opportunities to determine if BAT provides cardiometabolic benefits through redox homeostasis beyond thermogenesis.

### Limitation of the study:

We are aware of two limitations: First, we need to determine the extent to which BAT-derived metabolites, relative to other tissue sources, affect liver function. For example, the liver is estimated to be the primary source of circulating glutathione, accounting for approximately 80% of circulating glutathione levels in mice ^57^. Thus, reduced circulating glutathione levels in MBC^UCP1^ KO mice likely reflect reduced glutathione levels in the liver rather than a consequence of reduced glutathione release from the BAT. Meanwhile, previous studies reported that impaired antioxidant systems in adipocytes, such as the fat-specific deletion of glutathione peroxidase 4 (GPX4) or glutamate-cysteine ligase (GCLC), led to insulin resistance ^58,59^. Second, the present human study is limited to healthy young men with limited sample size. Future studies are warranted to investigate the correlation between BAT activity and circulating metabolites in larger cohorts.

## STAR Methods

### RESOURCE AVAILABILITY

#### Lead Contact

Further information and requests for resources and reagents should be directed to and will be fulfilled by the lead contact, Shingo Kajimura (skajimur@bidmc.harvard.edu).

#### Materials availability

Mouse strains and plasmids generated in this study are available upon request from the lead contact.

#### Data and code availability

- For proteomic datasets the MS raw data and analysis files were deposited with the ProteomeXchange Consortium (http://proteomecentral.proteomexchange.org) ^60^. The dataset identifier for TurboID proteomics (Figure 3) is PXD044020 and the dataset identifier for mitochondrial proteome (Figure 6B) is PXD043992. The phosphoproteome MS raw data and analysis files have been deposited with the ProteomeXchange Consortium via the jPOST partner repository (https://jpostdb.org) with the data set identifier PXD043813. Metabolomic dataset LC-MS raw data are uploaded to the Metabolomics Workbench (https://www.metabolomicsworkbench.org), with a project ID PR001873 and a project DOI 10.21228/M8MF0Z. The datasets are publicly available.
- This paper does not report original code.
- Any additional information required to reanalyze the data reported in this work paper is available from the lead contact upon request

### EXPERIMENTAL MODEL AND STUDY PARTICIPANT DETAILS

#### Mouse strains and husbandry

All animal experiments conducted were performed in compliance of protocols approved by the Institutional Animal Care and Use Committee at Beth Israel Deaconess Medical Center (028–2022). All mice were housed under a 12 h – 12 h light/dark cycle. Room-temperature mice were housed at 23°C in ventilated cages with an ACH of 25. Mice housed at thermal neutral conditions were housed in an incubator at 30°C. Mice exposed to cold were individually housed in an incubator set to 4–12°C. Starting at 6–8 weeks of age mice were fed a standard diet (Lab Diet 5008) or high-fat diet (Research Diets; D12492; 60% Fat) and had free access to food and water, unless experimentally specified. Experiments in standard diet fed mice were conducted in adult aged mice after 8–10 weeks of age. For mice fed HFD, metabolic experiments were conducted after 8 weeks and up until 16 weeks of HFD feeding, unless otherwise stated. Wild-type mice were purchased from Jackson Laboratory (Strain # 000664) and given one week of acclimation prior to any experimental intervention. All genetic models used littermates as controls. Inhibition of the mitochondrial BCAA carrier (MBC, *Slc25a44*) in all tissues was achieved using CRISPR-dCas9 fused to Krüppel associated box (KRAB) transcriptional repressor domain, as reported ^24^. BAT-specific deletion of the MBC was achieved using the Cre-lox system with *Ucp1*-Cre, as reported ^27^. For glutathione supplementation studies, buffers were carefully brought to pH 6.8 for administration to mice, as glutathione is labile at a pH over 7 ^61^. Mice supplemented with glutathione (Sigma G4251) received a daily *i.p.* injection of 2g/kg/d at pH 6.8 for 13 days. Mice were treated with BSO (0.445 mg/g/d) by *i.p.* injection for up to 35 days. No sex-specific differences were observed between control and MBC-KD mice. Male mice were used unless otherwise stated.

#### Cells culture

All cell culture experiments were performed with immortalized brown adipocytes ^27^. The base media for brown adipocyte cell culture was DMEM (Gibco 11965092), containing 10% FBS and 1% penicillin/streptomycin (Gibco 15140). For virus production, HEK293T packaging cells were transfected with 10 μg of retroviral or lentiviral plasmid and the packaging constructs through calcium phosphate method. To induce differentiation in brown adipocytes, confluent preadipocytes were treated with an induction cocktail consisting of 0.5 mM isobutylmethylxanthine (Sigma I5879), 125 nM indomethacin (Sigma I7378), 2 μg/mL dexamethasone (Sigma D4902), 850 nM insulin (Sigma I6634), and 1 nM T3 (Sigma T2877) for 2 days. Following induction, the cells were switched to a maintenance medium of base medium supplemented with 850 nM insulin and 1 nM T3. The cells were kept in the maintenance medium for 4–6 days to achieve full differentiation. To inhibit BCAT2 activity pharmacologically, we used BCAT-IN-2 at 50 μM ^34^ and Telmisartan at 100 μM ^35^.

#### Human serum analyses

Healthy adult East Asian (Japanese) male volunteers (N = 33) underwent positron emission tomography-computed tomography with ^18^F-fluorodeoxyglucose (^18^FDG-PET/CT) following 2-hour cold exposure at 19°C. The protocols were approved by the Institutional Research Ethics Review Board of Tenshi College in Sapporo, Japan (Protocol# 2015–25) and the University of Tokyo, Japan (Protocol# 23–82). Information on the subjects is provided in Table S2. The subjects were separated into high- and low-BAT groups based on the presence or absence of radioactivity greater than that of the background (SUV < 2.0) in the supraclavicular adipose tissue Subjects with SUV ≥ 2.0 were considered as the high BAT group with SUV < 2.0 were the low BAT group in the study ^62^. After fasting for ~12 h, serum was collected from subjects rested at 27°C. Subjects were then exposed to 19°C, after 2 hours at 19 °C serum was collected. Serum samples were assessed for total glutathione using commercially available kit (Abcam, ab205811).

### METHOD DETAILS

#### Plasmids and virus production

Cells expressing *Slc25a44* tagged with Flag sequence were previously generated ^24^. The *Slc25a44* or *Bcat2* cDNA sequence was amplified and cloned in-frame with Turbo-ID into the retroviral expression vector (Addgene, 75085). For shRNA mediated gene knockdown, shRNA targeting *Slc25a22* (TRCN0000069108), and BCAT2 (sh1-GTGGACGTTACACTCCAAAGC; sh2-GCACCATGAACATCTTTGTCT) were generated and scrambled shRNA were acquired from (Genecopoeia, CSHCTR001-LVRU6GH). The shRNA targeting control scrambled, *Slc25a22* or *Bcat2* were cloned into PLKO.1-blasticidin (Addgene, 26655). All constructs were confirmed by sequencing. For virus production HEK293T packaging cells were transfected with 10 μg of retroviral or lentiviral plasmid and the packaging constructs through calcium phosphate method. After 48 h, the viral supernatant was collected and filtered through a 0.45 μm filter. Immortalized preadipocytes were infected with viral supernatant supplemented with 10 μg ml^−1^ polybrene for 24 h. Stable cell lines were selected with blasticidin (10 μg ml^−1^).

#### Metabolite extraction

In order to maintain the inherent metabolome, tissues were immediately snap frozen in liquid nitrogen and stored at −80°C until processing. For cell culture, cold extraction buffer was added to cells over dry ice. When tissues or cells were homogenized, the samples were processed on ice or at 4°C. Plasma metabolites were extracted using a 1:4 ratio of plasma to methanol extraction buffer. After centrifugation at 16,000 g for 15 minutes at 4°C, the supernatant was collected for LC-MS analysis. Brown fat metabolites were extracted by homogenizing the tissues with 80% methanol at a 40:1 volume to wet weight ratio. To extract metabolites from adipocytes, the tracing media was aspirated and the cells were immediately incubated with 500 μL of cold methanol containing 1μg/ml of the internal standard (D8-Phe) for 5 minutes on dry ice. The cells were then scraped into Eppendorf tubes and homogenized in a TissueLyser II (Qiagen) for 15 minutes at 30 Hz at 4 °C. Next, 200 μL of the extract was mixed with 100 μL of Milli-Q water and 200 μL of chloroform. The mixture was then centrifuged at 16000g for 5 minutes at 4 °C. Subsequently, 150 μL of the aqueous solution was filtered through a 10-kDa cut-off filter (MRCPRT010, Millipore) to remove proteins. The filtrate was transferred to the glass insert for LC-MS detection. To extract metabolites from media samples, the cell debris was removed by centrifuging the medium at 16,000 x g for 5 min at 4 °C. Then, 200 μL of clarified medium was transferred to the Eppendorf tube prefilled with 800 μL of cold methanol. After mixing for 30 seconds, the samples were incubated at −80°C for 1 hour and then centrifuged at 16,000 x g at 4°C for 15 minutes. Finally, 50 μL of the supernatant was transferred to the glass insert for LC-MS detection.

#### Extracellular fluid isolation

Brown adipose tissue and epidydimal white adipose tissue were dissected from adult wild-type male and female mice (Jax; 000664) and placed in the center of 20 μm nylon net filter (Millipore, NY2004700), which was secured in a 1.5 mL tube ^32,63^. BAT was then centrifuged at 800 x g for 10 minutes at 4°C. The extracellular fluid collected from the centrifugation was snap-froze in liquid nitrogen and stored in −80°C until metabolite extraction. Metabolites were extracted from the extracellular fluid using a ratio of 1:20 (EF: Extraction buffer). Extracellular fluid extraction buffer consisted of 80% methanol containing inosine-^15^N_4_, thymine-d4, and glycocholate-d4 internal standards. Metabolite intensity was normalized to the average of internal standards.

#### ^15^N-BCAA and ^13^C-BCAA tracing in cells

To determine the metabolic fate of BCAA-nitrogen in mouse brown adipocytes, we used ^15^N-Leu, ^15^N-Ile, and ^15^N-Val as tracers. The tracers were added to a BCAA and glutamine-free high glucose DMEM containing dialyzed FBS (10%) at a concentration of 1.6 mM each. Tracing studies were also conducted using ^15^N-Leu or ^15^N-Val individually under the same conditions. In addition, we conducted a ^15^N-BCAA tracing study in BCAA-free high glucose DMEM supplemented with 2 mM glutamine. To prepare unlabeled media, we added 1.6 mM of each unlabeled Leu, Ile, and Val to high glucose DMEM with 10% dialyzed FBS. Cell media was replaced with fresh unlabeled media 12 hours before the isotope switch. After switching to the tracing media, we collected media and cell samples at 0 h, 6 h, 12 h, 24 h, and 36 h. For ^13^C-BCAA tracing studies, we added 1.6 mM of each ^13^C-Leu (CLM-2262-H-0.25), ^13^C-Ile (CLM-2248-H-0.25), and ^13^C-Val (CLM-2249-H-0.25) to the BCAA and glutamine-free high glucose DMEM containing dialyzed FBS (10%). The media and cell samples were collected at 0 h, 2 h, 6 h, and 24 h after switching to the tracer media. The Compound Discoverer 3.3 (Thermo Fisher Scientific) was used to analyze the percentage of labeled BCAA and related metabolites.

#### ^15^N-BCAA tracing in mice

Male C57BL/6J mice, 8 weeks old, with jugular vein catheters were purchased from The Jackson Laboratory. The mice were fed a chow and HFD diet for 8 weeks. The mouse infusion setup (Instech Laboratories) included a tether and swivel system that allowed the animals to move freely in the cage. Before infusion, mice were fasted for 4 hours. A mixture of ^15^N-Leu, ^15^N-Ile, and ^15^N-Val in saline was infused via the catheter at a constant rate of 0.0836 μL/g/min. The tracer infusion rates for ^15^N-Val, ^15^N-Leu, and ^15^N-Ile were 2.5, 3.0, and 1.3 nM/min/g, respectively. These doses were selected to minimize perturbation of BCAA homeostasis ^23^. The concentration of each ^15^N-BCAA in the mixture tracing solution was determined by their infusion rates. Approximately 20 μL of blood was collected from the tail at 0 h, 2 h, 4 h, 8 h, and 12 h into tubes containing clotting activator (Starstedt Inc, 16.440.100). The blood samples were kept on ice and the serum was separated by centrifugation at 3,000 g for 10 min at 4 °C. At the end of the tracing experiment, each set of animals was sacrificed by cervical dislocation, and the indicated tissues were immediately harvested and snap frozen. The circulating fluxes of BCAA were calculated using the formula Fcirc = R*(1 - L)/L, where R represents the constant infusion rate and L represents the labeling percentage at steady state.

For bolus tracing studies in MBC-KD and paired control mice, a mixture of ^15^N-BCAA (75 μg in total/g of body weight, Val: Leu: Ile = 1: 1.5: 0.8) was administered via i.p. injection following 4 hours of fasting following the previous study ^64^. For the study of mass-action, a mixture of ^15^N-Leu, ^15^N-Ile, and ^15^N-Val (50, 100, and 200 in total μg/g of body weight, Val: Leu: Ile = 1: 1.5: 0.8) was i.p. injected to male C57BL/6J mice at 10 weeks old at equal volume.

#### LC-MS metabolomics

Metabolomics data were acquired using a UHPLC system (Vanquish Horizon, Thermo Scientific) coupled to an orbitrap mass spectrometer (Exploris 240, Thermo Scientific). Waters ACQUITY UPLC BEH Amide column (particle size, 1.7 μm; 100mm (length) × 2.1mm (i.d.)) was used for LC separation. The column temperature was kept at 25 °C. Mobile phases A = 25mM ammonium acetate and 25mM ammonium hydroxide in 100% water, and B = 100% acetonitrile, were used for negative mode. The linear gradient eluted from 95% B (0.0–1 min), 95% B to 65% B (1–7.0 min), 65% B to 40% B (7.0–8.0 min), 40% B (8.0–9.0 min), 40% B to 95% B (9.0–9.1 min), then stayed at 95% B for 5.9 min. The flow rate was 0.4 mL/min. The sample injection volume was 2 μL for cell and 5 μL for media. ESI source parameters were set as follows: spray voltage, 3500 V or −2800 V, in positive or negative modes, respectively; vaporizer temperature, 350 °C; sheath gas, 50 arb; aux gas, 10 arb; ion transfer tube temperature, 325 °C. The full scan was set as: orbitrap resolution, 60,000; maximum injection time, 100 ms; scan range, 70–1050 Da. The ddMS2 scan was set as: orbitrap resolution, 30,000; maximum injection time, 60 ms; top N setting, 6; isolation width, 1.0 m/z; HCD collision energy (%), 30; Dynamic exclusion mode was set as auto. The ^15^N labeling metabolomics was quantified by Compound Discoverer 3.3. The annotation of metabolites was performed by searching the retention time and MS2 against our in-house library which was generated using the Mass Spectrometry Metabolite Library (MSMLS^™^) (IROA Technologies LLC^®^, Bolton, MA, USA). Phenylalanine-d8 was used as internal standard to evaluate the system variation. Peak areas were normalized using the internal standard and the relative changes were analyzed.

Metabolites were measured in mouse plasma using MRM-based LC-MS metabolite profiling techniques as previously described ^13,65^. Briefly, Hydrophilic interaction liquid chromatography/positive ion mode MS detection to measure polar metabolites are conducted using an LC-MS system comprised of Agilent 1260 Infinity HPLC coupled to 4000-QTRAP mass spectrometer (Sciex). Plasma samples (10 μL) are prepared via protein precipitation with the addition of nine volumes of 74.9:24.9:0.2 v/v/v acetonitrile/methanol/formic acid containing stable isotope-labeled internal standards (valine-d8, Sigma-Aldrich; St. Louis, MO; and phenylalanine-d8, Cambridge Isotope Laboratories; Andover, MA). The samples are centrifuged (20 min, 15,000 x g, 4°C), and the supernatants are injected directly onto a 150 × 2 mm, 3 μm Atlantis HILIC column (Waters). The column is eluted isocratically at a flow rate of 250 μL/min with 5% mobile phase A (10 mM ammonium formate and 0.1% formic acid in water) for 0.5 minute followed by a linear gradient to 40% mobile phase B (acetonitrile with 0.1% formic acid) over 10 minutes. MS analyses are carried out using electrospray ionization in the positive ion mode using scheduled MRM method. Multiquant software (version 3.0.3, Sciex) was used for automatic peak integration followed by manual review of all peaks for quality of integration. Central metabolites including sugars, sugar phosphates, organic acids, purine, and pyrimidines, are extracted from 30 μL of plasma using acetonitrile and methanol and separated using a 100 × 2.1 mm 3.5-μm Xbridge amide column (Waters). Mobile phase A was 95:5 (v/v) water/acetonitrile, with 20 mM ammonium acetate and 20 mM ammonium hydroxide (pH 9.5). Mobile phase B was acetonitrile. Tandem MS analysis for negative mode detection utilizes a high sensitivity Agilent 6490 QQQ mass spectrometer equipped with an electrospray ionization source. The settings were as follows: sheath gas temperature, 400°C; sheath gas flow, 12 l/min; drying gas temperature, 290°C; drying gas flow, 15 l/min; capillary, 4,000 V; nozzle pressure, 30 psi; nozzle voltage, 500 V; and delta EMV, 200 V. Raw data are processed using MassHunter Quantitative Analysis Software (Agilent).

#### Cellular respirometry

Oxygen consumption rate (OCR) in fully differentiated brown adipocytes was measured with the Seahorse XFe Extracellular Flux Analyzer (Agilent) in a 24-well plate. For the experiments in Figure 3, the experimental respiration media was Hanks balanced salt solution (HBSS) supplemented with 1 mM pyruvate, 2.5 mM glucose, and 1% BSA. In order, BCAA (2 mM) and norepinephrine (10 μM) were injected to sample wells. Data are normalized to the baseline average for comparative analysis. For Figure S2, the experimental media was KRB-HEPES buffer supplemented with 15 mM glucose, 200 nM adenosine, and 2% BSA. The experimental media was first injected with 2 mM Valine, 2 mM KIV, or vehicle. Subsequently, the experimental media was injected with 10 μM norepinephrine. Data are normalized to protein amount.

#### BCAA oxidation

Wild-type male C57BL/6J mice (Jax; 000664) at 8 weeks of age were randomly placed into diet intervention groups of standard chow, 4 weeks HFD feeding, or 12 weeks of HFD feeding. Mice in the 4 weeks of HFD feeding were fed a standard diet for the first 8 weeks of the study and switched to HFD for the final 4 weeks. After the diet intervention, food was removed for 4 h prior to tissue collection. From euthanized mice, brown adipose tissue (BAT), inguinal white adipose tissue (Ing-WAT), heart, kidney, and liver were collected for BCAA oxidation. Approximately 50 mg of tissue was used for the assay. Tissue was incubated in modified KRP buffer (15 mM glucose, 20 mM HEPES, 0.5 mM MgCl_2_, 120 mM NaCl, 4.56 mM KCl, 0.7 mM Na_2_HPO_4_, 1.3 mM NaH_2_PO_4_, 200 nM adenosine, 1 mM leucine, 2% bovine serum albumin) with [1-^14^C] leucine (American Radiolabeled Chemicals). Tissues in the reaction buffer were rotated for 30 minutes at 37°C. To stop the reaction, 300 μL of 30% H_2_O_2_ was added to the mix and capped with a smear containing 1 M benzethonium hydroxide. Samples were incubated at room temperature for 20 minutes, and the smear was added to scintillation vials. Scintillation fluid was added, and samples were counted for radioactivity.

#### BCAA tolerance test

Experiment was conducted as previously described ^24^. Mice were single housed and fasted for the experiment. Mice received a single bolus of BCAA via oral gavage and placed in cold chamber set to 12°C. BCAA were delivered at a ratio by weight of 1:1.5:0.8 (Val:Leu:Ile). Tail blood was collected at indicated time. Serum BCAA were measured with commercially available kit (Abcam, ab83374).

#### Affinity purification and proteomics

Brown adipocytes expressing MBC-FLAG or empty-vector controls were fully differentiated using the method detailed under “Cell Culture” of STAR Methods. Mitochondrial were enriched from cells using a sucrose gradient isolation^66^. Mitochondria were homogenized in buffer containing 200 mM sucrose, 10 mM HEPES, 1 mM EGTA, 1 mg/mL fatty acid-free BSA at pH 7.2. Cell homogenate was centrifuged at 800 x g for 5 min at 4°C and supernatant was centrifuged and at 10000 x g for 10 min at 4°C to pellet mitochondria. Enriched mitochondrial fraction was suspended in lysis buffer (25 mM Tris pH 7.4, 150 mM NaCl, 1 mM EGTA, 1% n-dodecyl β-D-maltoside (DDM)) supplemented with proteinase inhibitor (Roche, cOmplete). Mitochondrial lysates were normalized to protein content for immunoprecipitation. Samples were incubated in IP buffer containing 50 mM Tris pH 7.4, 150 mM NaCl, 1 mM EGTA, 0.5 mM DTT, 15% glycerol, and 0.01% DDM and precleared with Protein G (Cytiva) for 2 hours. Next, we incubated samples with FLAG-beads (Thermo Fisher A36798) overnight. Samples were then washed 2x with IP buffer and then 10x with wash buffer (50 mM Tris pH 7.4, 150 mM NaCl). Sample was then eluted off beads using 3X-FLAG peptide. We performed TCA precipitation on the eluted samples and separated proteins on a 12% SDS-PAGE gel. Gel bands were excised and were then subjected to a modified in-gel trypsin digestion procedure ^67^. Gel pieces were washed and dehydrated with acetonitrile for 10 min, followed by acetonitrile removal. Pieces were then completely dried in a speed-vac. Rehydration of the gel pieces was with 50 mM ammonium bicarbonate solution containing 12.5 ng/μl modified sequencing-grade trypsin (Promega, Madison, WI) at 4ºC. After 45 min., the excess trypsin solution was removed and replaced with 50 mM ammonium bicarbonate solution to just cover the gel pieces. Samples were then placed in a 37ºC room overnight. Peptides were later extracted by removing the ammonium bicarbonate solution, followed by one wash with a solution containing 50% acetonitrile and 1% formic acid. The extracts were then dried in a speed-vac (~1 h). The samples were then stored at 4ºC until analysis. On the day of analysis, the samples were reconstituted in 5 – 10 μl of HPLC solvent A (2.5% acetonitrile, 0.1% formic acid). A nano-scale reverse-phase HPLC capillary column was created by packing 2.6 μm C18 spherical silica beads into a fused silica capillary (100 μm inner diameter x ~30 cm length) with a flame-drawn tip ^68^. After equilibrating the column, each sample was loaded via a Famos autosampler (LC Packings, San Francisco CA) onto the column. A gradient was formed, and peptides were eluted with increasing concentrations of solvent B (97.5% acetonitrile, 0.1% formic acid). As peptides eluted, they were subjected to electrospray ionization and then entered into an LTQ Orbitrap Velos Pro ion-trap mass spectrometer (Thermo Fisher Scientific, Waltham, MA). Peptides were detected, isolated, and fragmented to produce a tandem mass spectrum of specific fragment ions for each peptide. Peptide sequences (and hence protein identity) were determined by matching protein databases with the acquired fragmentation pattern by the software program, Sequest (Thermo Fisher Scientific, Waltham, MA) ^69^. All databases include a reversed version of all the sequences, and the data was filtered to between a one and two percent peptide false discovery rate. Proteins with at least two unique, valid peptides were included in further analyses.

#### Turbo-ID proximity labeling and proteomics

Fully differentiated brown adipocytes expressing empty vector, MBC-TurboID, or BCAT2-TurboID were used for the experiments. Cells were incubated with 50 μM biotin for 30 minutes. Then biotin containing media was aspirated, cells were washed and subsequently incubated for four hours. After the four-hour biotin washout period, cells were washed with ice-cold phosphate-buffered saline (PBS), and cells were collected. Next, we isolated mitochondria using differential centrifugation detailed in “Affinity purification and proteomics”, and normalized samples to mitochondrial protein for pulldown of biotinylated proteins. Samples were incubated overnight with streptavidin beads (Cytiva, Streptavidin Mag Sepharose), and then washed 5 minutes for each of the following steps: 2x (2% SDS), 1x (50 mM HEPES pH 7.4, 500 mM NaCl, 1 mM EDTA, 0.1% deoxycholate, 1% Triton-X 100), 1x (10 mM Tris pH 7.4, 250 mM LiCl, 1 mM EDTA 0.5% NP-40, 0.5% deoxycholate), 2x (50 mM Tris pH 7.4, 50 mM NaCl). Samples were then resuspended (50 mM Tris pH 7.4, 50 mM NaCl) and stored at −80°C until on bead digestion. For on bead digestion, beads were washed with 50 mM Tris (pH 8.0) buffer, followed by resuspension in 1M Urea, 50 mM Tris (pH 8.0) and initially digesting it with 2 μg Trypsin (Promega) at 37°C for two hours. After initial trypsin incubation, samples were centrifuged briefly, and the supernatants were collected in new tubes. Beads were further washed three times with 1M Urea, 50 mM Tris (pH 8.0); washed fractions were pooled with supernatants and left to digest overnight at room temperature. The following morning, digested peptides were reduced first with 5 mM TCEP, followed by alkylation with 10 mM Iodoacetamide, quenching alkylation with 5 mM DTT, and finally, quenching the digestion process with TFA. Acidified digested peptides were desalted over C18 StageTips ^70^. Peptides were then eluted with 80% acetonitrile, 0.1% TFA and dried in a speed vac. Dried samples were reconstituted with 200 mM EPPS buffer, pH 8.0, and labeled with TMTPro reagents (Thermo Fisher Scientific). Following incubation at room temperature for 2 h, the reactions were quenched with hydroxylamine to a final concentration of 0.5% (v/v). Samples were combined, further desalted over StageTips, and finally eluted into autosampler inserts (Thermo Scientific), dried in a speedvac, and reconstituted with 5% Acetonitrile, 5% TFA for MS analysis. Mass spectrometric data were collected on an Orbitrap Eclipse mass spectrometer (with a FAIMS device enabled) coupled to a Thermo Easy-nLC 1000. The 100 μm capillary column was packed in-house with 35 cm of Accucore 150 resin (2.6 μm, 150Å; ThermoFisher Scientific). The peptides were separated using a 180 min linear gradient from 5% to 32% buffer B (90% ACN + 0.1% formic acid) equilibrated with buffer A (5% ACN + 0.1% formic acid) at a flow rate of 550 nL/min across the column. The scan sequence began with an MS1 spectra were collected in the Orbitrap (resolution - 60,000; scan range - 400−1,600 m/z; automatic gain control (AGC) target – 400,000; maximum injection time – automatic). MS2 spectra were collected in the Orbitrap following higher-energy collision dissociation (resolution – 50,000; AGC target – 125,000; normalized AGC target - 250%; NCE (normalized collision energy) – 36; isolation window - 0.5 Th; maximum injection time - 86ms). FAIMS compensation voltages (CVs) were set at - 40V, −60V, and −80V.

#### Mitochondrial proteomics

Isolated mitochondrial proteomes were reduced with 5 mM TCEP, alkylated with 10 mM Iodoacetamide, further reduced with 5 mM DTT, and precipitated using TCA (final concentration of 20%). The precipitated samples were washed three times with ice-cold acetone. Proteins were solubilized in a digestion buffer (1M urea, 200 mM EPPS pH 8.5) and digested with Lys-C overnight. The samples were further digested with trypsin for 6 hours at 37°C for six hours. The digested samples were labeled with TMTPro reagents (Thermo Fisher Scientific). Following incubation at room temperature for 2 hours, the reactions were quenched with hydroxylamine to a final concentration of 0.5% (v/v). Following TMT labeling, the samples were combined, and the pooled sample was de-salted using a Sep-pak. To perform mitochondrial TMT proteomics, labeled peptides were fractionated using Pierce High pH Reversed-Phase Peptide Fractionation Kit (Thermo Scientific). A total of 6 fractions were collected. Samples were subsequently acidified with 1% formic acid and vacuum centrifuged to near dryness. Each consolidated fraction was desalted by StageTip, and reconstituted in 5% acetonitrile, 5% formic acid for LC-MS/MS analysis. Data were collected on an Orbitrap Fusion Lumos Tribird mass spectrometer (Thermo Fisher Scientific) equipped with a Thermo Easy-nLC 1000 for online sample handling and peptide separations. The 100 μm capillary column was packed in-house with 35 cm of Accucore 150 resin (2.6 μm, 150Å; ThermoFisher Scientific). The peptides were separated using a 180 min linear gradient from 5% to 32% buffer B (90% ACN + 0.1% formic acid) equilibrated with buffer A (5% ACN + 0.1% formic acid) at a flow rate of 550 nL/min across the column. Data was collected using an SPS-MS3 method. The scan sequence for the Fusion Lumos Orbitrap began with an MS1 spectrum collected in the Orbirap (resolution - 120,000; scan range - 350 – 1,500 m/z; AGC target – 1,000,000; normalized AGC target – 250%; maximum ion injection time 50 ms; dynamic exclusion - 180 seconds). MS2 spectra were collected in the ion trap following collision-induced dissociation (AGC target – 15,000; normalized AGC target - 150%; NCE (normalized collision energy) – 35; isolation window - 0.5 Th; maximum injection time - 50ms). MS3 scans were collected in the Orbitrap following higher-energy collision dissociation (resolution – 50,000; AGC target – 100,000; normalized AGC target – 200%; collision energy – 55%; MS2 isolation window – 2; number of notches – 10; MS3 isolation window – 1.2; maximum ion injection time – 200 ms.

#### Proteomics data analyses

Database searching included all entries from the mouse UniProt Database (downloaded in May 2021). The database was concatenated with one composed of all protein sequences for that database in the reversed order ^71^. Raw files were converted to mzXML, and monoisotopic peaks were re-assigned using Monocle ^72^. Searches were performed with Comet ^73^ using a 50-ppm precursor ion tolerance and fragment bin tolerance of 0.02. TMTpro labels on lysine residues and peptide N-termini +304.207 Da), as well as carbamidomethylation of cysteine residues (+57.021 Da) were set as static modifications, while oxidation of methionine residues (+15.995 Da) was set as a variable modification. Peptide-spectrum matches (PSMs) were adjusted to a 1% false discovery rate (FDR) using a linear discriminant after which proteins were assembled further to a final protein-level FDR of 1% analysis ^74^. TMT reporter ion intensities were measured using a 0.003 Da window around the theoretical m/z for each reporter ion. Proteins were quantified by summing reporter ion counts across all matching PSMs. More specifically, reporter ion intensities were adjusted to correct for the isotopic impurities of the different TMTpro reagents according to manufacturer specifications. Peptides were filtered to exclude those with a summed signal-to-noise (SN) < 160 across all TMT channels and < 0.5 precursor isolation specificity. The signal-to-noise (S/N) measurements of peptides assigned to each protein were summed (for a given protein).

#### Glucose and insulin tolerance

MBC-KD mice as well as littermate controls fed with high-fat diet were housed at room temperature (23°C). MBC^UCP1^ KO mice and littermate controls were fed high-fat diet and housed at thermoneutrality (30°C). Mice were fasted for 4 hours prior to intraperitoneal delivery of glucose (1g per kg body mass) or insulin (1 U per kg body mass). Blood glucose from the tail vein was measured repeatedly from 0 to 120 minutes post-injection with a handheld glucometer (Abbott, Freestyle Lite). For glucose-stimulated insulin secretion measurement, serum was collected during GTT at 0 minutes before glucose injection and at 15 minutes post-injection of glucose. Serum insulin was measured with a commercially available kit (Millipore, EZRMI-13K).

#### *In vivo* insulin signaling

Mice were fasted for 4 hours and then were delivered an intraperitoneal injection of insulin at 1 U per kilogram body mass. After 12 minutes, the animal was sacrificed, and the liver, brown adipose tissue, inguinal white adipose tissue, and soleus were rapidly dissected and snap-frozen in liquid nitrogen and stored at −80°C until further processing. Tissues were homogenized in lysis buffer supplemented with protease (Roche, cOmplete protease) and phosphatase inhibitor cocktails (Sigma, P5726, P0044). The lysis buffer consisted of 150 mM NaCl, 5 mM EDTA, 1% Triton-X 100, 0.1% sodium deoxycholate, and 0.1% SDS. The tissue lysates were separated by SDS-PAGE and immunoblotted for total-AKT and phospho-AKT^Ser473^ (Cell Signaling 4691, Cell Signaling 4060).

#### Phosphoproteomics

The experiment was performed following the method employed previously ^75^. In brief, liver tissue was rapidly dissected from mice 12 minutes after an i.p. injection of insulin, as described under *in vivo* insulin signaling method. Liver samples were homogenized in lysis buffer supplemented with protease and phosphatase inhibitors. Trypsin digestion was conducted according to eFASP protocol ^76^. After high-pH reversed-phase fractionation into 6 fractions by StageTip as described above, each fraction was analyzed by TiO2-based hydroxy acid-modified metal oxide chromatography (HAMMOC) ^77^ to enrich phosphopeptides. The above procedure was performed on 14 different liver tissue samples (wild type with insulin treatment n=6, wild type without insulin treatment n=1, MBC-KD with insulin treatment n=6 and MBC-KD without insulin treatment n=1) for a total of 84 phosphopeptide samples. Each of the 14 samples from the same high pH RP fraction was then labeled with a different isobaric tag using 14 of Thermo TMTpro *16plex* label reagent Sets ^78^. After mixing the 14 TMT-labeled samples, 6 phosphopeptide samples were desalted with StageTips for subsequent nanoLC-MS/MS analysis using a Thermo Orbitrap Fusion Lumos connected to a Thermo Ultimate 3000 pump and an HTC-PAL autosampler (CTC Analytics). Phosphopeptides were loaded onto in-house needle columns (150-mm length, 100 μm inner diameter, 6-μm needle opening) packed with ReproSil-Pur 120 C18-AQ 3-μm RP material (Dr Maisch) through a 5-μL loop. The flow rate was 500 nL/min. Separation was achieved by applying a three-step linear gradient of 4 to 8% ACN in 5 min, 8 to 32% ACN in 60 min, 32 to 80% ACN in 5 min, and 80% ACN for 5 min with 0.5% acetic acid. Spray voltage was set to 2.4 kV, the ion transfer tube was heated to 250 °C. MS1 spectra were collected at a resolution of 120,000. Data-dependent Orbitrap MS2 scans were collected in the Top Speed mode using a cycle time of 3 s between Full MS scans. The quadrupole isolation width was set to 0.7 Th. The Orbitrap was operated at 50,000 resolution, and precursors were fragmented by high-energy collision dissociation (HCD) at a normalized collision energy (NCE) of 38%. Phosphoproteome data were analyzed using MaxQuant against UniProt Mouse Database with a strict trypsin/p specificity allowing for up to 2 missed cleavages. Carbamidomethyl (C) was set as a fixed modification. Oxidation (M), acetyl (protein N-term) and phosphorylation (STY) were allowed as variable modifications. The resulting evidance.txt file was used for the analysis. For the data analyses, the sample without insulin treatment (n=1 each group) was removed because of insufficient number for statistical analyses. For insulin-treated samples, there were significant variations among the samples, likely due to suboptimal sample conditions. One sample from KO mice was removed from the analysis as it showed above±1SD variation for >50% of molecules analyzed. Accordingly, the data analyses were based on n=6 from the control and n=5 from KO mice using unpaired t-test.

#### Pyruvate dehydrogenase activity

Insulin-stimulated (see in vivo insulin signaling) whole-tissue lysate was used for the assay. One hundred micrograms of tissue lysates were used in a commercially available kit (Abcam, ab109902).

#### Indirect calorimetry

Male MBC^UCP1^ KO and littermate control mice fed standard chow diet were singly housed and monitored with Promethion Metabolic Cage System (Sable Systems). Mice were initially housed at thermoneutrality (30°C) and then exposed to a cold challenge (4°C) before returning to room temperature (23°C). Mice had unrestricted access to food and water throughout the experiment. The average light and dark cycle measurements were calculated by taking the last full light or dark cycle prior to the temperature being changed. Data were analyzed with CalR (https://calrapp.org/) ^79^.

#### Absolute metabolite abundance

Absolute quantification of serum BCAA from mice was measured with a commercially available kit (Abcam, ab83374). The assay was measured after 30 minutes of incubation at room temperature and serum volume used ranged from 1 – 6 μL per sample. Absolute quantification of glutathione in serum or tissue homogenates was performed with a commercially available kit (Abcam, ab205811). Tissue samples were homogenized using 0.5% NP-40 in PBS at pH 6.0 to avoid spontaneous oxidation of glutathione. Assay was incubated at room temperature for 15–30 minutes. All values were corrected through blank subtraction prior to quantification using standard curve.

#### Oxidative stress markers

Protein oxidative stress accumulation in the liver was determined through protein carbonyl content, which was measured using a commercially available kit (Abcam, ab126287). Assay was performed with 500 μg of liver protein and counts were normalized to protein content in assay through BCA assay. Lipid oxidative stress in the liver was determined by measuring malondialdehyde (MDA) with commercially available kit (Abcam, ab118970) utilizing colormetric assay and 6.67 mg of tissue, and through immunoblotting for 4-hydoxy-2-noneal (4-HNE; Abcam, ab48506) with densitometry quantification of band at 50 kDa.

#### Lipid measurement

Serum and liver triglycerides were measured using a commercial kit (Thermo Scientific, TR2241). As previously described for liver ^80^, ~50 mg of tissue was homogenized in 350 μL of ethanolic KOH (2 ethanol: 1 KOH) and incubated in 55°C for 8 hours. Lysates were then volume up to 1 mL with 50% ethanol. Samples were vortexed and centrifuged at 15,000 rpm at room temperature for 5 minutes. After centrifugation, 200 μL of the supernatant was incubated with 215 μL of 1 M MgCl_2_ on ice for 10 minutes. Samples were centrifuged at 15,000 rpm at 4°C, and the supernatant was used for the measurement of triglyceride content. Lipidomics of liver samples were performed at the Duke University School of Medicine Proteomics and Metabolomics Core Facility.

#### Quantitative RT-PCR (qPCR)

Total RNA was isolated from cells or tissue using Trizol (Invitrogen) according to manufacturer instructions. RNA was reverse transcribed using iScript cDNA synthesis kit (Biorad). PCR reactions were performed with Applied Biosystems QuantStudio 6 Flex using Sybrgreen (Biorad). Assays were performed in duplicate, and all results were normalized to 18S ribosomal RNA or 36B4, which was unchanged between controls and respective experimental groups. All values are relative to the mean of the control group. Primers used are listed in Table S3.

#### Quantification And Statistical Analysis

Statistics were performed using GraphPad Prism software. Figure 1H statistic used was t-test with unequal variance. Figure 2 used 2-way ANOVA with Šídák’s multiple comparisons test for all panels. Figure 3A used a two-tailed Pearson correlation to compare BCAA catabolism proteins. Figure 3D used 2-way ANOVA with Dunnett’s multiple comparisons test. Figure 3E used one-way ANOVA with Dunnett’s multiple comparisons test. Figure 4A, 4D, and 4F used unpaired t-test. Figure 4B, 4G, 4H, 4K, and 4L used 2-way ANOVA with Šídák’s multiple comparisons test, and AUC statistic is unpaired t-test. Figure 4C used 2-way ANOVA with Šídák’s multiple comparisons test. Figure 4E and 4J used multiple unpaired t-test with multiple comparisons corrected by two-stage step-up (Benjamini, Krieger, and Yekutieli) method. Figure 4I used ANCOVA with total body mass as covariate. Figure 5A, 5B, 5C, 5D, 5E, 5F, 5I, and 5K used unpaired t-test. Figure 5G, 5H, and 5J used 2-way ANOVA with Šídák’s multiple comparisons test, and AUC statistic is unpaired t-test. Figure 5L used two-tailed Pearson correlation. Figure 6A used one-way ANOVA with Dunnett’s multiple comparisons test. Figure 6B, 6D, 6E, 6F, and 6G used unpaired t-test. Figure 6H used Wilcoxon matched-pairs signed rank test with multiple comparisons corrected by two-stage step up (Benjamini, Krieger, and Yekutieli) method to compare between 27°C and 19°C and Mann Whitney test to compare high BAT at 19°C to low BAT at 19°C. Figure 6I used two-tailed Pearson correlation. Bars represent group mean and error shown as s.e.m.

## Supplementary Material

1 Figure S1. BCAAs are key nitrogen donors in brown adipocytes, related to Figure 1 **A.** Relative abundance of indicated metabolites in the extracellular fluid from wild-type male and female mice BAT and epidydimal Epi-WAT. Metabolite counts in extracellular fluid are normalized to internal control signal. N = 12 per group (6 male, 6 female). Statistic: unpaired t-test.
**B.** Time course of ^15^N-BCAA derived metabolites in control brown adipocytes. Cells were incubated with ^15^N-BCAA for 36 hours. Data are shown as mean with s.e.m. N = 3 per group.
**C.** ^15^N labeling percentage of nitrogen metabolites in the absence or presence of glutamine (0.5 mM, 1 mM, and 2 mM) in cultured brown adipocytes. Differentiated brown adipocytes were cultured with ^15^N-BCAA (1.6 mM each) for 24 hours. Labeled metabolites are presented as percent M+1. N = 3 per metabolite.
**D.** ^15^N labeling percentage of nitrogen metabolites using ^15^N-Val (left) and ^15^N-Leu (right) in brown adipocytes. Differentiated brown adipocytes were cultured with 1.6 mM tracer for 24 hours. Labeled metabolites are presented as percent M+1. N = 3 per metabolite.

2 Figure S2. MBC is required for the synthesis of BCAA-derived metabolites, related to Figure 2 **A.** Representative western blot of MBC protein abundance in control and MBC KO brown adipocyte mitochondria with TOM20 as mitochondrial protein loading control.
**B.** ^15^N-BCAA labeled metabolites (M+1) in whole-cell and isolated mitochondria from control or MBC KO adipocytes. Brown adipocytes were cultured with ^15^N-BCAAs for 24 hours and metabolites were extracted from whole-cell or enriched mitochondrial fraction. Data represented as z-score heat map for each metabolite with each cell representing quantitated value. N = 3 per group for whole cell and 4 per group for mitochondrial fraction. Statistic: 2-way ANOVA with Šídák’s multiple comparisons test.
**C.** Norepinephrine induced ^15^N-Valine derived metabolites in control and MBC KO brown adipocytes. Values are shown as ^15^N-labeled metabolite intensity normalized to control group mean. N = 9 per group. Statistic: unpaired t-test.
**D.** Labeled metabolites in the media of control and MBC KO brown adipocyte. Following incubation with ^15^N-BCAA for 24 hours, media was analyzed for ^15^N-labeled metabolites. M+0 and M+1 labeled metabolites are stacked per genotype and are relative to control group. N = 3 per group. Statistic: 2-way ANOVA with Šídák’s multiple comparisons test.
**E.** mRNA expression of *Bcat2* in differentiated brown adipocytes stably expressing shRNAs targeting BCAT2 (shRNA-*Bcat2* #1 and #2) or a scrambled control. N = 4 per group. Statistic: unpaired t-test.
**F.** ^15^N-labelled Glu levels in brown adipocytes expressing a scrambled control shRNA and shRNA-BCAT2. Cells were incubated with ^15^N-BCAA for 2 hours. N = 3 per group. Statistic: 2-way ANOVA with Šídák’s multiple comparisons test.
**G.** ^13^C-labelled BCKA and indicated TCA intermediates in control and MBC KO brown adipocytes following incubation with ^13^C-labelled BCAA for 2 hours. Data represented as z-score heat map for each metabolite with each cell representing quantitated value. N = 3 per group. Statistic: t-test with two sample unequal variance. *p<0.05, **p<0.01, ***p<0.001.
**H.** Norepinephrine (NE) stimulated OCR in control and MBC KO brown adipocytes. Cells were treated with vehicle, Val, or keto-isovalerate (KIV) and stimulated with NE. Mean NE-induced change in OCR from baseline shown to the right of time course. N = 7 for vehicle and valine, N = 6 for keto-isovalerate. Statistic: One-way ANOVA with Dunnett’s multiple comparisons test.
**I.** Relative levels of indicated glycolytic intermediates in control and MBC KO brown adipocytes. Data represented as z-score heat map for each metabolite with each cell representing quantitated value. N = 3 per group. Statistic: t-test with two sample unequal variance. *p<0.05, **p<0.01, ***p<0.001.

3 Figure S3. MBC associated proteome in brown fat, related to Figure 3 **A.** Venn Diagram of mitochondrial proteins detected from MBC-flag pulldown (blue) and MBC-TurboID proximity labeling (red) in brown adipocytes. Identical mitochondrial proteins found in both methods are in grey. MBC-flag pulldown proteins were affinity purified, gel resolved, excised, digested with trypsin, and analyzed with reverse-phase liquid chromatography with tandem mass spectrometry (LC-MS/MS). MBC-TurboID proximity labeling proteins were affinity purified and subjected to tandem mass tag (TMT) proteomic quantification. Identified proteins were crossed referenced with MitoCarta 3.0 and assessed for identical proteins between methods.
**B.** BCAA catabolic pathway map. Proteins identified in both MBC-flag pulldown and MBC-TurboID proteomic methods are shown in bold. Proteins detected in only one method (HIBCH, MBC-flag pulldown; HMGCL, MBC-flag pulldown; MUT, MBC-TurboID) are un-bolded.
**C.** Gene ontology of biological processes from the 284 proteins detected in both MBC-flag pulldown and MBC-TurboID proteomic methods. Red bars represent BCAA pathways. Data are represented as fold enrichment.
**D.** *Gc1 (Slc25a22)* mRNA levels in indicated brown adipocytes. N = 4 per group. Statistic: One-way ANOVA with Dunnett’s multiple comparison’s test.
**E.** mRNA levels of genes in BCAA metabolic pathway in MBC-KO and control brown adipocytes. N = 3 per group. Statistic: two-way ANOVA with Šídák’s multiple comparisons test.
**F.** Unlabeled (M+0) metabolites in brown adipocytes incubated with ^15^N-BCAA for 24 hours. Data are relative to control group. N = 3 per group. Statistic: one-way ANOVA with Dunnett’s multiple comparisons test.

4 Figure S4. Impaired BCAA flux caused insulin resistance independent of energy expenditure, related to Figure 4 **A.** *Slc25a44* mRNA levels in indicated tissues from control and MBC-KD male mice. N = 5 per group per tissue. Statistic: unpaired t-test.
**B.** Dose response of ^15^N-BCAAs in circulation in wild-type male mice over 30 minutes. Mice were given an intraperitoneal injection of BCAAs at a low (50 μg g^−1^ body mass), medium (100 μg g^−1^), or high (200 μg g^−1^) dose. N = 8 per dose.
**C.** Mass action of BCAA and BCAA-derived metabolites in circulation 10 minutes after *i.p.* injection of ^15N^BCAA at a dose of 50, 100, or 200 μg per g body mass. N = 8 per dose, male mice. Statistic: coefficient of determination (R^2^).
**D.** Tissue weights of high fat diet fed male control and MBC-KD, blue) male mice. N = 5 for control and 6 for MBC-KD. Statistic: unpaired t-test.
E. Inguinal white adipose tissue mRNA of thermogenic markers from control and MBC-KD male mice. N = 5 per group. Statistic: unpaired t-test.
**F.** Serum triglyceride (TG) levels in fasting control and MBC-KD male mice fed high fat diet. N = 7 per group. Statistic: unpaired t-test.
**G.** Serum insulin levels in high fat diet fed control and MBC-KD male mice under fasting conditions (0 minutes) and 15 minutes after i.p. glucose delivery (glucose stimulated insulin secretion, GSIS). N = 7 per group. Statistic: 2-way ANOVA with Šídák’s multiple comparisons test.
**H.** Body mass of female control and female MBC-KD mice on a high fat diet. N = 8 control and 11 MBC-KD. Statistic: multiple unpaired t-test with multiple comparisons corrected by two-stage step-up (Benjamini, Krieger, and Yekutieli) method.
**I.** Glucose tolerance test and area under the curve (AUC) of high fat diet fed female control and female MBC-KD mice. Mice were fasted for 4 hours prior to collecting baseline blood glucose measurement and subsequent intraperitoneal injection of glucose (1 g kg^−1^ body mass). Glucose induced changes in blood glucose were recorded through 120 minutes post glucose delivery. N = 5 control mice and 6 for MBC-KD mice. Statistic for insulin tolerance curve is 2-way ANOVA with Šídák’s multiple comparisons test and AUC statistic is unpaired t-test.
**J.** *Slc25a44* mRNA levels in indicated tissues of control MBC^UCP1^ KO male mice. N = 3 per group per tissue. Statistic: unpaired t-test.
**K.** Energy expenditure per 12-hour light cycle at each temperature intervention in control and MBC^UCP1^ KO male mice fed standard chow diet. N = 4 for control mice and 3 for MBC^UCP1^ KO mice. Statistic: ANCOVA with total body mass as covariate.
**L.** Cold tolerance test during adaptation to 4°C. Changes in rectal temperature of control and MBC^UCP1^ KO male mice were recorded. N = 5 for both groups. Statistic: 2-way ANOVA with Šídák’s multiple comparisons test.
**M.** Food intake per 12-hour light cycle at each temperature intervention in (B). Statistic: 2-way ANOVA with Šídák’s multiple comparisons test.
**N.** Activity per 12-hour light cycle at each temperature intervention in (B). Statistic: 2-way ANOVA with Šídák’s multiple comparisons test.
**O.** Body composition of control and MBC^UCP1^ KO male mice fed high fat diet for 10 weeks. Mice were weighed and placed in EchoMRI to assess fat mass and lean mass. N = 8 per group. Statistic: unpaired t-test.

5 Figure S5. BAT-specific MBC deletion impaired insulin signaling in the liver, related to Figure 5 **A.** *In vivo* insulin stimulation of AKT ^Ser473^ phosphorylation in indicated tissues. Mice were fasted 4 hours prior to insulin injection and rapid tissue excision. Western blots of phospho-AKT^Ser473^ over total-AKT. N = 6 per group per tissue, male mice.
**B.** Triglyceride content of livers from high fat diet fed control and MBC^UCP1^ KO mice. N = 6 per group. Statistic: unpaired t-test.
**C.** Liver contents of indicated lipid species in control and MBC^UCP1^ KO male mice on a high-fat diet. N = 8 per group. Statistic: unpaired t-test.
**D.** Western blot of lipid peroxidation marker 4-hydoxynonenal (4-HNE) from liver tissue of control and MBC^UCP1^ KO male mice fed high fat diet. N = 6 per group.
**E.** Liver glutathione levels prior (left) and post supplementation of glutathione (right) in control and MBC^UCP1^ KO male mice. N = 6 per group prior and 7 for control and 6 for MBC^UCP1^ KO mice post. Statistic: unpaired t-test.
**F.** Serum glutathione levels post-supplementation of glutathione in (A). Statistic: unpaired t-test.
**G.** Glutathione levels in the serum (left) and liver (right) of wild-type male mice. Mice on a high-fat diet were supplemented with GSH (2g kg^−1^ d^−1^) or vehicle for 10 days. N = 10 for vehicle control and 9 for GSH supplementation. Statistic: unpaired t-test.
**H.** Body weight changes in wild-type male mice in (C). Statistic is multiple unpaired t-test with multiple comparisons corrected by two-stage step-up (Benjamini, Krieger, and Yekutieli) method.
**I.** Insulin tolerance test and area under the curve (AUC) of wild-type male mice supplemented with glutathione or vehicle. Mice were fasted for 4 hours prior to collecting baseline blood glucose measurement and subsequent intraperitoneal injection of insulin (0.8 U kg^−1^). N = 10 for vehicle control and 9 for GSH supplementation. Statistic for insulin tolerance curve is 2-way ANOVA with Šídák’s multiple comparisons test, and AUC statistic is unpaired t-test.
**J.** *In vivo* insulin stimulation of AKT^Ser473^ phosphorylation in liver of control and MBC^UCP1^ KO male mice post supplementation of glutathione. Mice were fasted 4 hours prior to insulin injection and rapid tissue excision. Data are quantification of western blot analysis of phospho-AKT^Ser473^ over total-AKT. N = 6 per group per tissue. Statistic: unpaired t-test.
**K.** Body weight changes in wild-type male mice during BSO treatment. Mice on a regular diet were supplemented with BSO (0.445 mg g^−1^ d^−1^) or vehicle for 35 days. N = 9. Statistic is multiple unpaired t-test with multiple comparisons corrected by two-stage step-up (Benjamini, Krieger, and Yekutieli) method.

6 Figure S6. Pathophysiological regulation of BCAA catabolism in BAT, related to Figure 6 **A.** Body mass of wild-type male mice fed a standard diet, short-term high fat diet (4 weeks), or long-term high fat diet (12 weeks). N = 6 per group. Statistic: 2-way ANOVA with Šídák’s multiple comparisons test.
**B.** Fasting blood glucose of mice in (A). Statistic: One-way ANOVA with Dunnett’s multiple comparisons test.
**C.** *Ex vivo* ^14^C-leucine oxidation in indicated tissues of mice (A). N = 6 per group. Statistic: One-way ANOVA with Dunnett’s multiple comparisons test.
**D.** BCAA catabolic pathway map. Proteins identified as downregulated by high fat diet feeding, as shown in Figure 6B, are in blue bolded text.
**E.** Circulating disposal rate (Rd, nmol min^−1^ g^−1^) of ^15^N-Leu, ^15^N-Val, and ^15^N-Ile in lean and obese mice. Male C57BL/6 mice with jugular vein catheters were fed a chow and high-fat diet for 8 weeks. A mixture of ^15^N-Leu, ^15^N-Ile, and ^15^N-Val in saline was infused via the catheter at a constant rate of 0.0836 μl/g/min. N = 4 per group. Statistic: unpaired t-test.
**F.** Changes in indicated ^15^N-labeled metabolites in the pancreas of male mice fed a high-fat diet relative to mice fed a regular diet. Tissues were collected from mice stably infused with ^15^N-BCAA for 12 hours. N = 4 per group. Statistic: unpaired t-test. Data shows labelling percentage of tissue nitrogen metabolites normalized to the labeling percentage of tissue BCAA, expressed as the percent difference in obese mice relative to lean mice.
**G.** Temperature induced changes in serum levels of BCAA-nitrogen derived metabolites N-acetylglutamate and glutathione. Serum was collected from wild-type male mice housed at room temperature (23°C). Serum was then collected after exposure to a 12-hour cold challenge at 6°C. N = 9 per group. Statistic: multiple paired t-test with two-stage step-up (Benjamini, Krieger, and Yekutieli) correction method. Line connects paired sample.

7

## Figures and Tables

**Table:** KEY RESOURCES TABLE

REAGENT or RESOURCE	SOURCE	IDENTIFIER
**Antibodies**		
Total-AKT	Cell Signaling	Cat # 4691
Phospho-AKT^Ser473^	Cell Signaling	Cat# 4060
4-hydroxy-2-noneal (4-HNE)	Abcam	Cat# ab48506
SLC25A44	Yoneshiro et al.^24^	N/A
TOM20	Proteintech	Cat# 11802-1-AP
Pierce^™^ Anti-DYKDDDDK Magnetic Agarose	Thermo Fisher Scientific	Cat# A36798
Streptavidin	Sigma-Aldrich	Cat# GE28-9857-38
Protein G	Cytiva	Cat# 17-0618-01
Goat anti-Mouse IgG (H+L) Secondary Antibody, HRP	Thermo Fisher Scientific	Cat# 31430; RRID:AB_10960845
Goat Anti-Rabbit IgG H&L (HRP)	abcam	Cat# ab6721; RRID:AB_955447
**Biological Samples**		
Human serum	This paper	N/A
**Chemicals, Peptides, and Recombinant Proteins**		
Indomethacin	Sigma-Aldrich	Cat# I7378
Insulin	Sigma-Aldrich	Cat# I6634
Isobutylmethylxanthine (IBMX)	Sigma-Aldrich	Cat# I5879
Dexamethasone	Sigma-Aldrich	Cat# D4902
3,3′,5-Triiodo-L-thyronine (T_3_)	Sigma-Aldrich	Cat# T2877
L-(−)-Norepinephrine(+)-bitartrate salt monohydrate	Sigma-Aldrich	Cat# A9512
DMEM	Gibco	Cat# 11965092
Fetal Bovine Serum	ATLANTA biologicals	Cat# S11550
Penicillin-Streptomycin	Gibco	Cat# 15140
Basticidin S HCl (10 mg/mL)	Gibco	Cat# A1113903
0.05% Trypsin	Corning	Cat# MT25052CI
cOmplete^™^, EDTA-free Protease Inhibitor Cocktail	Roche	Cat# 11873580001
Phosphatase inhibitor cocktail 2	Sigma-Aldrich	Cat# P5726
Phosphatase inhibitor cocktail 3	Sigma-Aldrich	Cat# P0044
Dextrose	Sigma-Aldrich	Cat# D9434
Glucose solution	Thermo Fisher Scientific	Cat# A2494001
Bovine Serum Albumin	Sigma-Aldrich	Cat# A1595
DMEM, no glutamine	Gibco	Cat# 11960044
15N-Leucine	Cambridge Isotope Laboratories	Cat# NLM-142-1
15N-Isoleucine	Cambridge Isotope Laboratories	Cat# NLM-292-0.25
15N-Valine	Cambridge Isotope Laboratories	Cat# NLM-316-0.5
Leucine	Sigma-Aldrich	Cat# L8000
Isoleucine	Sigma-Aldrich	Cat# I2752
Valine	Sigma-Aldrich	Cat# V0500
[1-^14C^]-Leucine	American radiolabeled chemicals	Cat# ARC 0156A
Glutathione	Sigma-Aldrich	Cat# G4251
Dialyzed FBS	Cytiva	Cat# SH30079.02
KIV	Thermo Fisher Scientific	Cat# 189720050
Glucose	Sigma-Aldrich	Cat# G8270
MgCl_2_	Sigma-Aldrich	Cat# M2393
NaCl	Sigma-Aldrich	Cat# S7653
KCl	Sigma-Aldrich	Cat# P9541
Na_2_HPO_4_	Fluka	Cat# 71639
NaH_2_PO_4_	Sigma-Aldrich	Cat# 71505
Adenosine	Sigma-Aldrich	Cat# A4036
30% H_2_O_2_	Sigma-Aldrich	Cat# H1009
Benzethonium hydroxide	Santa Cruz	Cat# sc-280610
Scintillation fluid	PerkinElmer	Cat# 6013326
Tris	Sigma-Aldrich	Cat# 11814273001
EGTA	Sigma-Aldrich	Cat# E4378
n-dodecyl β-D-maltoside	Sigma-Aldrich	Cat# D4641
DTT	Sigma-Aldrich	Cat# 43816
Glycerol	Sigma-Aldrich	Cat# G7793
3X-FLAG peptide	Sigma-Aldrich	Cat# F4799
Paraformaldehyde	Santa Cruz Biotechnology	Cat# SC281692
PBS	Gibco	Cat# 10010023
Phenylhydrazone	Sigma-Aldrich	Cat# C2920
Phosphate buffer solution	Thermo Fisher Scientific	Cat# P5244
Chloroform	Sigma-Aldrich	Cat# 650498
Acetonitrile	Thermo Fisher Scientific	Cat# A955
Methanol	Thermo Fisher Scientific	Cat# A456
D8-Phe	Cambridge Isotope Laboratories	Cat# DLM-372-1
inosine-^15^N_4_	Cambridge Isotope Laboratories	Cat# NLM-4264-0.01
thymine-d4	Cambridge Isotope Laboratories	Cat# DLM-1089-1
glycocholate-d4	Cambridge Isotope Laboratories	Cat# DLM-2742-0.01
ammonium acetate	Thermo Fisher Scientific	Cat# 60-020-13
ammonium hydroxide	Thermo Fisher Scientific	Cat# 60-023-92
Water LC/MS	Thermo Fisher Scientific	Cat# W64
formic acid	Thermo Fisher Scientific	Cat# A117
ammonium formate	Sigma-Aldrich	Cat# 09735
SDS	Thermo Fisher Scientific	Cat# AM9820
HEPES	Sigma-Aldrich	Cat# H43375
LiCl	Sigma-Aldrich	Cat# L4408
EDTA	Sigma-Aldrich	Cat# E5134
NP-40	Boston Bioproducts	Cat# P-877
Deoxycholate	Sigma-Aldrich	Cat# D6750
Triton-X 100	Thermo Fisher Scientific	Cat# BP151
Tween 20	Thermo Fisher Scientific	Cat# BP337
KOH	Boston Bioproducts	Cat# BZ-8038
BSA	Sigma-Aldrich	Cat# A7906
Pyruvate	Sigma-Aldrich	Cat# P4562
HBSS	Thermo Fisher Scientific	Cat# 14025092
XF calibrant solution	Agilent	Cat# 100840-000
**Critical Commercial Assays**		
Glutathione Detection Kit	Abcam	Cat# ab205811
Protein Carbonyl Content	Abcam	Cat# ab126287
PDH Activity	Abcam	Cat# ab109902
Malondialdehyde (MDA)	Abcam	Cat# ab118970
Triglyceride Reagent	Thermo Fisher Scientific	Cat# TR2241
Triglyceride Standard	Thermo Fisher Scientific	Cat# 23-666-422
iscript reverse transcription supermix for rt-qPCR	Bio-rad	Cat# 1708841
iTaq Universal SYBR Green Supermix	Bio-rad	Cat# 1725125
Biorad gels 4-20% 15 well	Bio-rad	Cat# 4561096
BCAA	Abcam	Cat# ab83374
Pierce^™^ BCA Protein Assay Kit	Thermo Fisher Scientific	Cat# 23225
Rat/Mouse Insulin ELISA	Sigma-Aldrich	Cat# EZRMI
XFe24 FluxPak	Agilent	Cat# 102340-100
Glucometer	Abbott	Cat# Freestyle Lite
Glucose strips	Abbott	Cat# 70827
Biorad gels 12% 10 well	Bio-rad	Cat# 4568044
**Deposited Data**		
TurboID proteomics	This paper	PXD044020
BAT mitochondrial proteomics	This paper	PXD043992
Phosphoproteomics	This paper	PXD043813
**Experimental Models: Cell lines**		
Immortalized brown adipocytes	Yoneshiro et al.^24^	N/A
*Slc25a44*^Flox/Flox^-Empty Vector immortalized brown adipocytes	Yoneshiro et al.^24^	N/A
*Slc25a44*^Flox/Flox^-Cre immortalized brown adipocytes	Yoneshiro et al.^24^	N/A
*Slc25a44*^Flox/Flox^-Empty Vector-scrambled immortalized brown adipocytes	This paper	N/A
*Slc25a44*^Flox/Flox^-Cre-scrambled immortalized brown adipocytes	This paper	N/A
*Slc25a44*^Flox/Flox^-Empty Vector-sh*Slc25a22* immortalized brown adipocytes	This paper	N/A
*Slc25a44*^Flox/Flox^-Cre-sh*Slc25a22* immortalized brown adipocytes	This paper	N/A
*Slc25a44*-FLAG immortalized brown adipocytes	This paper	N/A
*Slc25a44*-TurboID immortalized brown adipocytes	This paper	N/A
*Bcat2*-TurboID immortalized brown adipocytes	This paper	N/A
**Experimental Models: Organisms/Strains**		
Mouse: C57BL6J mice	Jackson Laboratory	Cat# 000664
Mouse: Slc25a44 gRNA	Yoneshiro et al.^24^	N/A
Mouse: dCas9/KRAB	Yoneshiro et al.^24^	N/A
Mouse: Slc25a44^Flox/Flox^	Yoneshiro et al.^27^	N/A
Mouse: *Ucp1*-Cre mice	Jackson Laboratory	Cat# 024670
**Oligonucleotides**		
A full list of qPCR primers in TableS3	This paper	N/A
**Software and Algorithms**		
CaIR-ANCOVA	Banks Lab	https://calrapp.org/
Compound Discoverer 3.3	Thermo Fisher Scientific	Cat# OPTON-3106
RStudio	R Core team	https://www.R-project.org/
heatmaps version 1.26.0	Bioconductor	https://bioconductor.org/packages/release/bioc/html/heatmaps.html
Biorender	Biorender	https://www.biorender.com/
MetaboAnalyst 6.0	MetaboAnalyst	https://www.metaboanalyst.ca/
Adobe Illustrator 2020	Adobe	https://www.adobe.com/products/illustrator.html
GO Enrichment Analysis	GeneOntology	http://geneontology.org/
GraphPad Prism 8	GraphPad	https://www.graphpad.com/scientific-software/prism/
**Other**		
Standard Diet	Lab Diet	Cat# 5008
High Fat Diet	Research Diets	Cat# D12492

## Highlights:

- BCAA is a key nitrogen source for synthesizing non-essential amino acids and GSH
- Mitochondrial BCAA import via MBC is required for BCAA-derived metabolite synthesis
- Reduced synthesis of BCAA-derived metabolites in BAT results in insulin resistance
- Obesity impairs BCAA nitrogen flux and BCAA-derived metabolite synthesis in BAT
