# Supplementary figures and images for: BCAA nitrogen flux in brown fat controls metabolic health independent of thermogenesis
Source: Cell. Author manuscript; Available in PMC 2024 Jun 3. (PMC11145561; doi:10.1016/j.cell.2024.03.030)

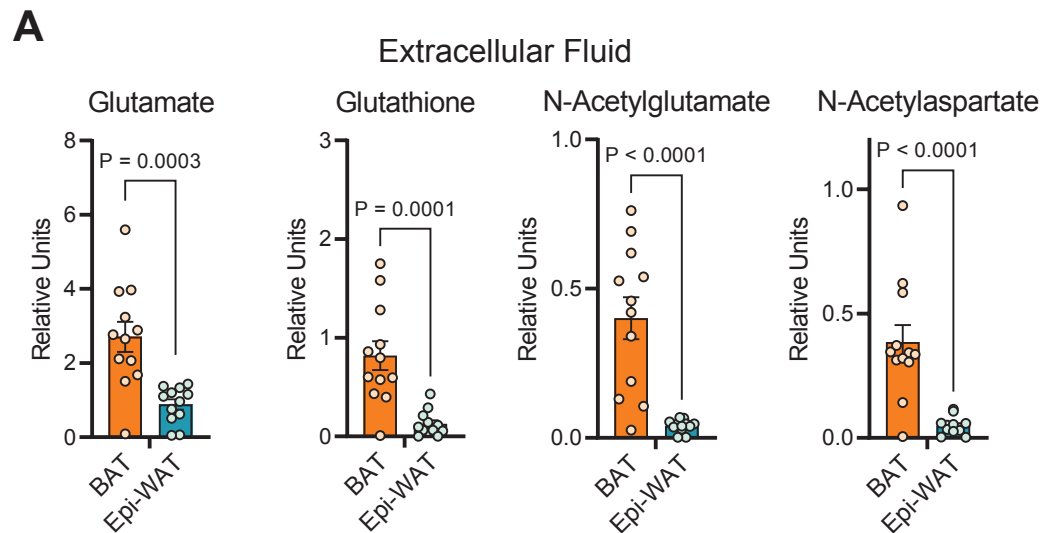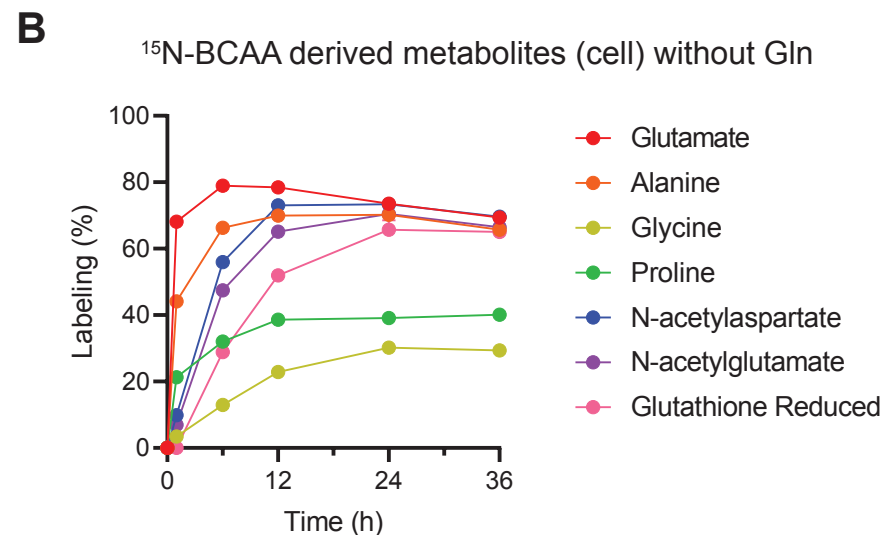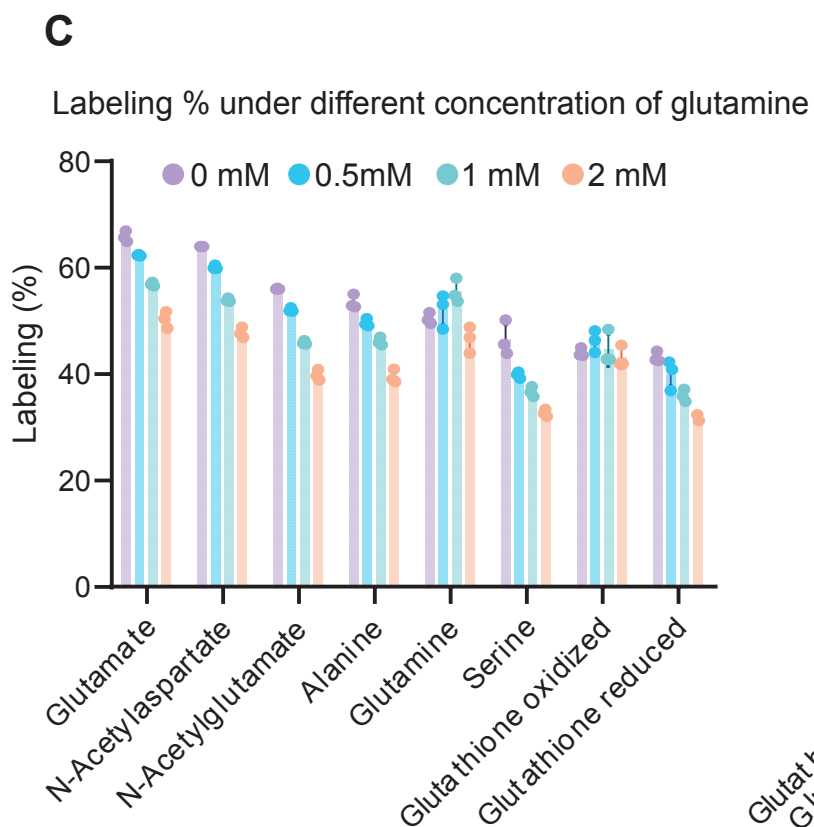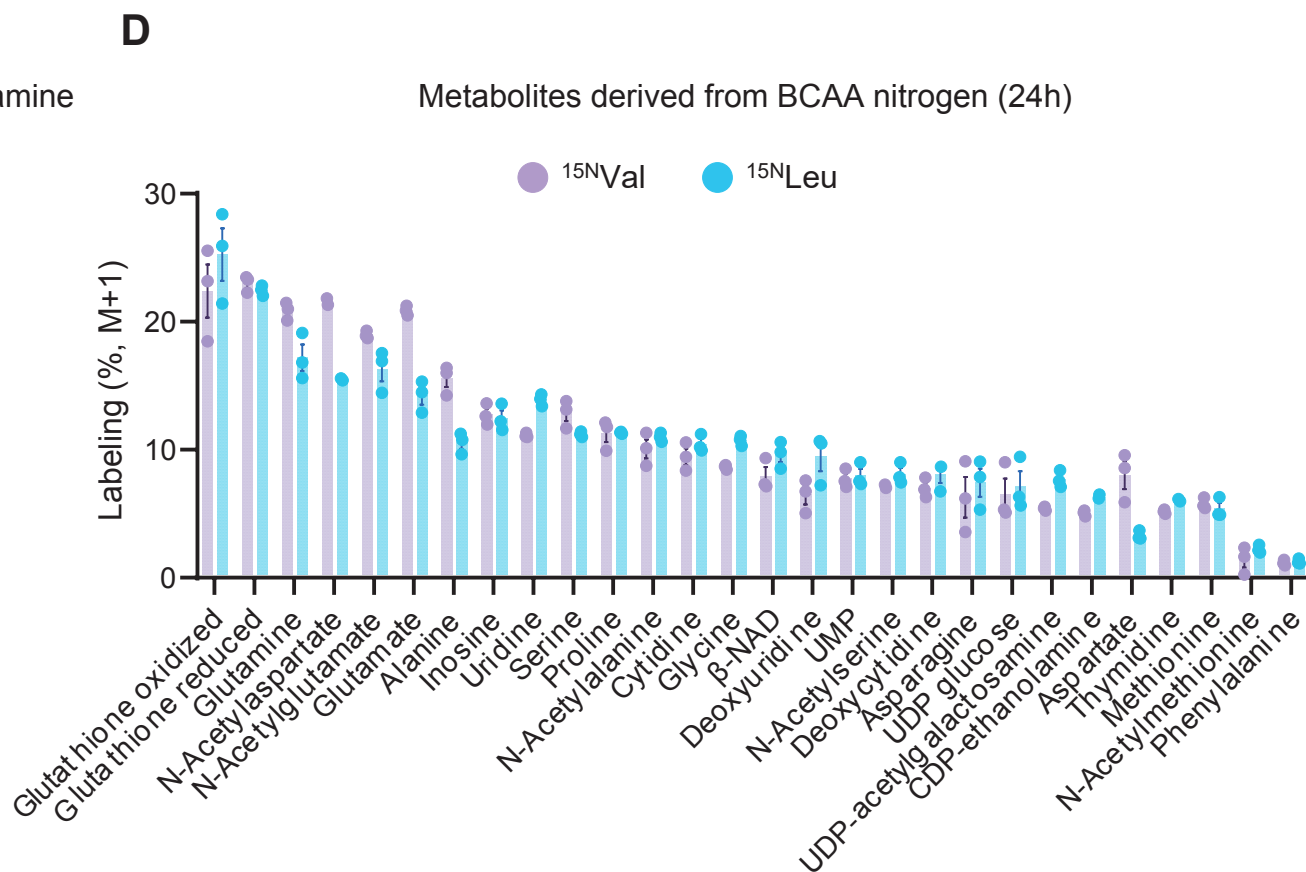

Supplement: 1 — A. Relative abundance of indicated metabolites in the extracellular fluid from wild-type male and female mice BAT and epidydimal Epi-WAT. Metabolite counts in extracellular fluid are normalized to internal control signal. N = 12 per group (6 male, 6 female). Statistic: unpaired t-test. B. Time course of 15N-BCAA derived metabolites in control brown adipocytes. Cells were incubated with 15N-BCAA for 36 hours. Data are shown as mean with s.e.m. N = 3 per group. C. 15N labeling percentage of nitrogen metabolites in the absence or presence of glutamine (0.5 mM, 1 mM, and 2 mM) in cultured brown adipocytes. Differentiated brown adipocytes were cultured with 15N-BCAA (1.6 mM each) for 24 hours. Labeled metabolites are presented as percent M+1. N = 3 per metabolite. D. 15N labeling percentage of nitrogen metabolites using 15N-Val (left) and 15N-Leu (right) in brown adipocytes. Differentiated brown adipocytes were cultured with 1.6 mM tracer for 24 hours. Labeled metabolites are presented as percent M+1. N = 3 per metabolite. [file NIHMS1982366-supplement-1.pdf]

**A**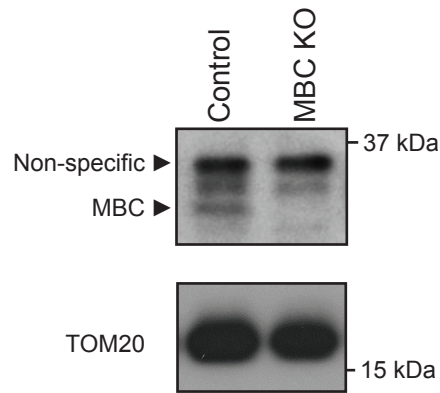**B**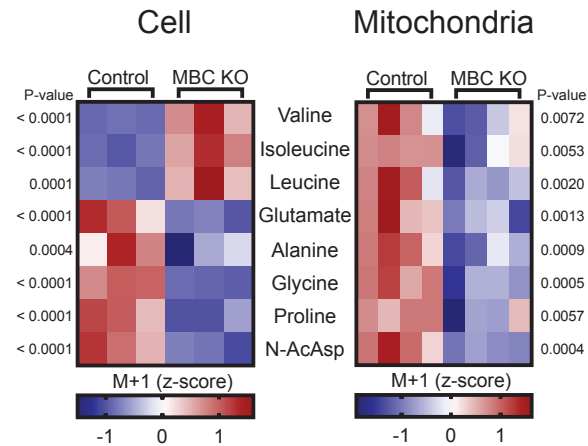**C**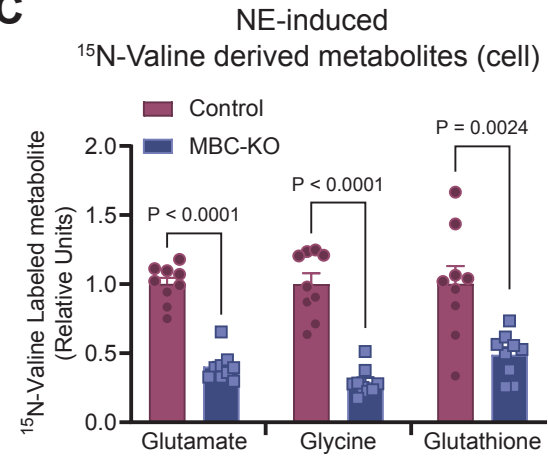**D**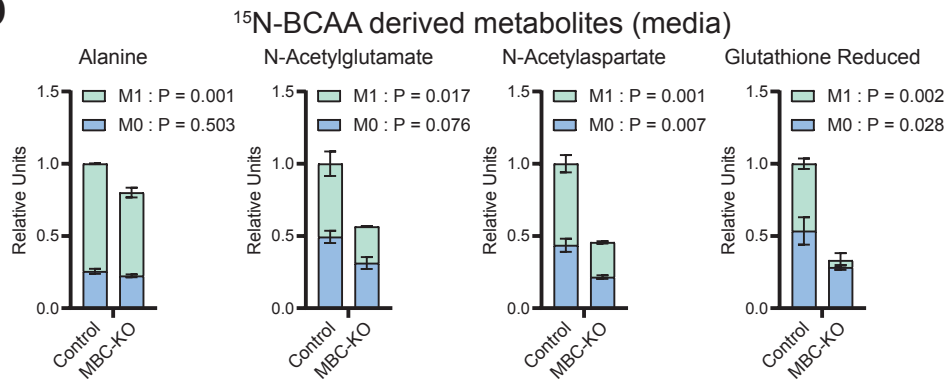**E**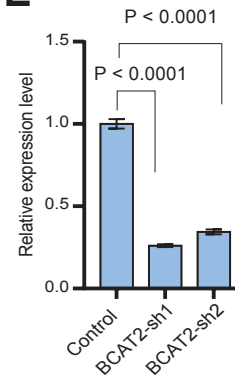**F**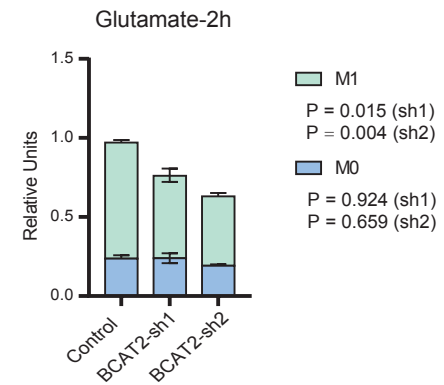**G**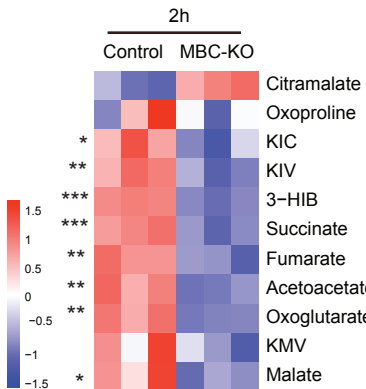**H**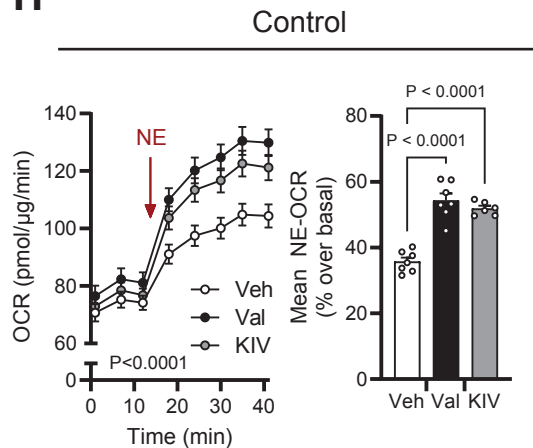**MBC KO**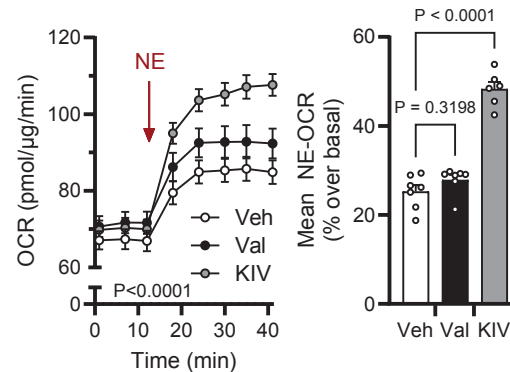**I****Glycolysis metabolites**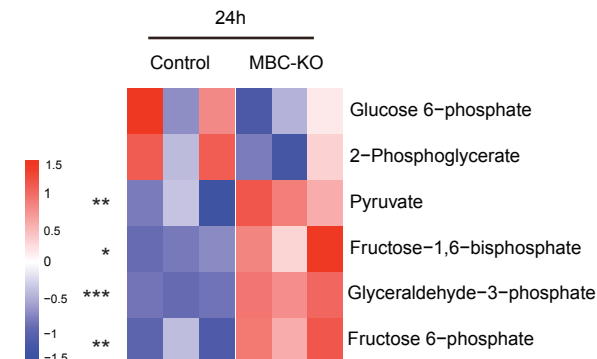

Supplement: 2 — A. Representative western blot of MBC protein abundance in control and MBC KO brown adipocyte mitochondria with TOM20 as mitochondrial protein loading control. B. 15N-BCAA labeled metabolites (M+1) in whole-cell and isolated mitochondria from control or MBC KO adipocytes. Brown adipocytes were cultured with 15N-BCAAs for 24 hours and metabolites were extracted from whole-cell or enriched mitochondrial fraction. Data represented as z-score heat map for each metabolite with each cell representing quantitated value. N = 3 per group for whole cell and 4 per group for mitochondrial fraction. Statistic: 2-way ANOVA with Šídák’s multiple comparisons test. C. Norepinephrine induced 15N-Valine derived metabolites in control and MBC KO brown adipocytes. Values are shown as 15N-labeled metabolite intensity normalized to control group mean. N = 9 per group. Statistic: unpaired t-test. D. Labeled metabolites in the media of control and MBC KO brown adipocyte. Following incubation with 15N-BCAA for 24 hours, media was analyzed for 15N-labeled metabolites. M+0 and M+1 labeled metabolites are stacked per genotype and are relative to control group. N = 3 per group. Statistic: 2-way ANOVA with Šídák’s multiple comparisons test. E. mRNA expression of Bcat2 in differentiated brown adipocytes stably expressing shRNAs targeting BCAT2 (shRNA-Bcat2 #1 and #2) or a scrambled control. N = 4 per group. Statistic: unpaired t-test. F. 15N-labelled Glu levels in brown adipocytes expressing a scrambled control shRNA and shRNA-BCAT2. Cells were incubated with 15N-BCAA for 2 hours. N = 3 per group. Statistic: 2-way ANOVA with Šídák’s multiple comparisons test. G. 13C-labelled BCKA and indicated TCA intermediates in control and MBC KO brown adipocytes following incubation with 13C-labelled BCAA for 2 hours. Data represented as z-score heat map for each metabolite with each cell representing quantitated value. N = 3 per group. Statistic: t-test with two sample unequal variance. *p<0.05, **p<0.01, *** [file NIHMS1982366-supplement-2.pdf]

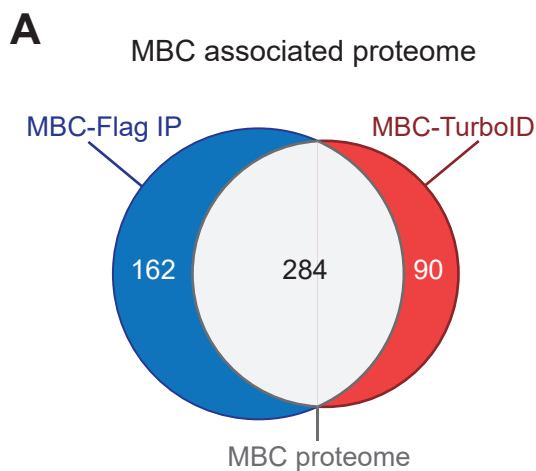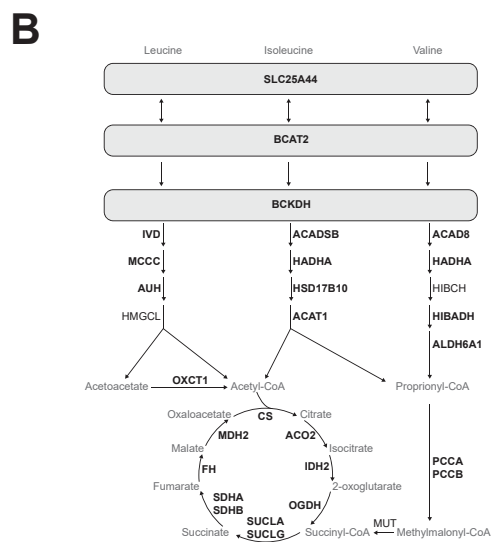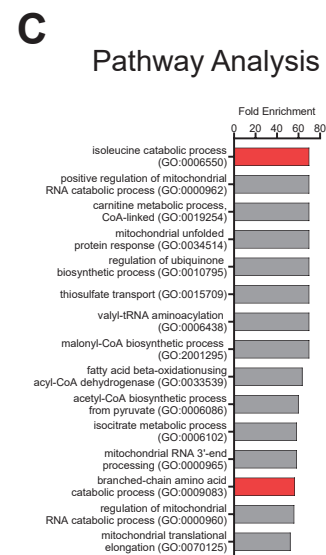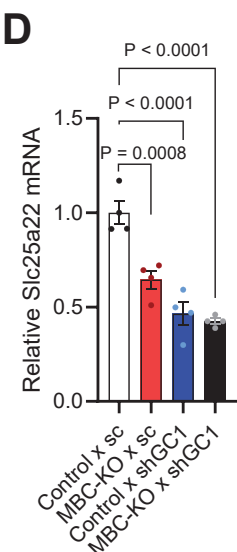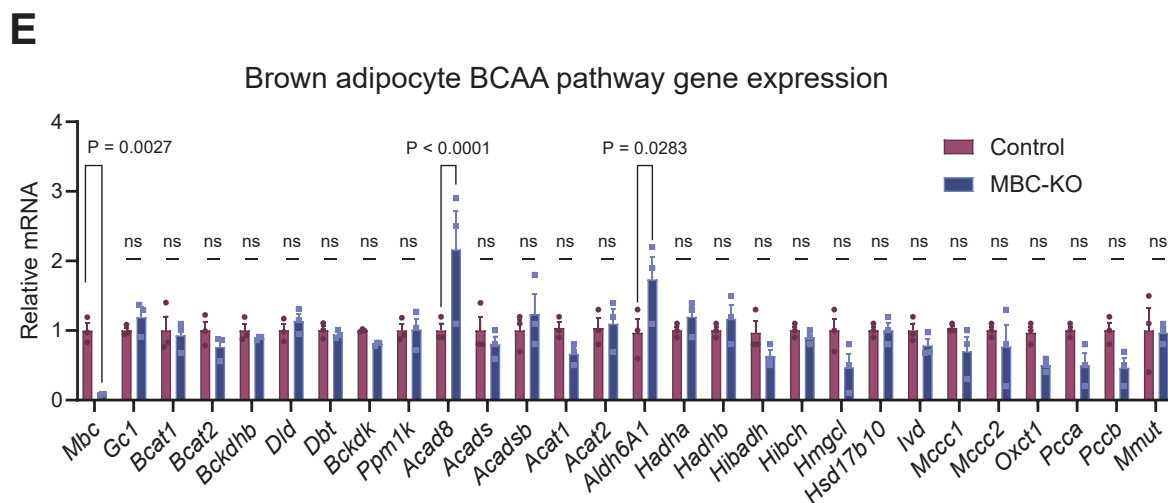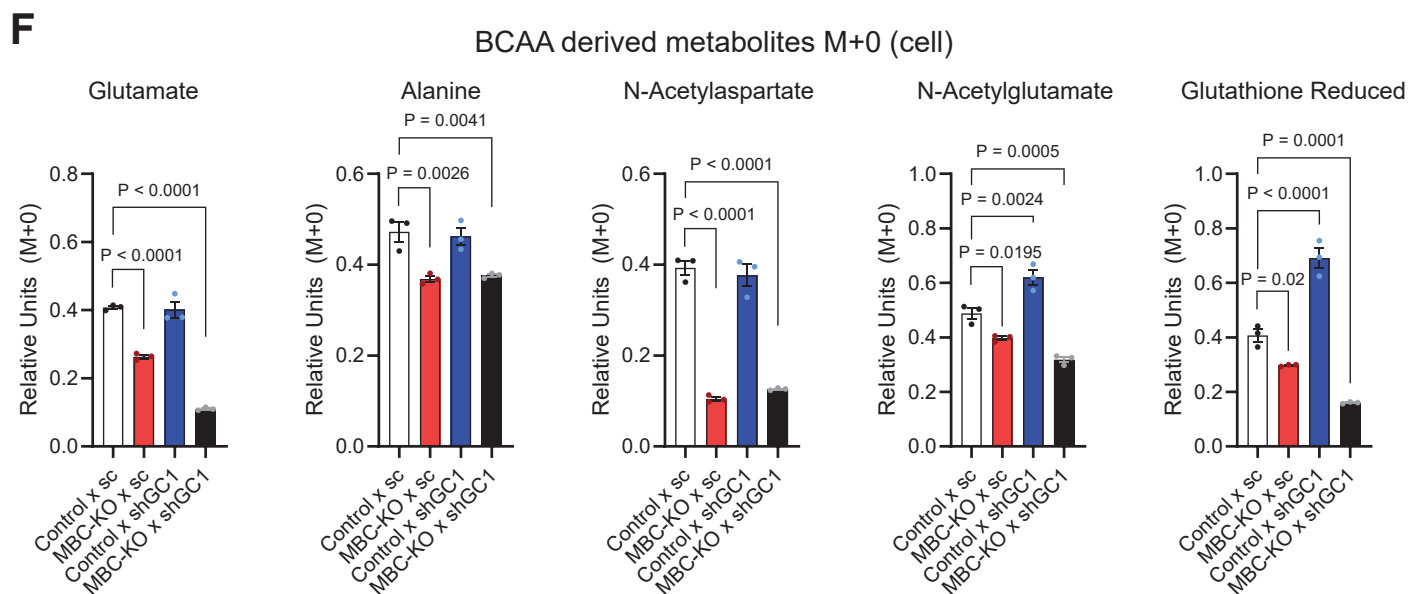

Supplement: 3 — A. Venn Diagram of mitochondrial proteins detected from MBC-flag pulldown (blue) and MBC-TurboID proximity labeling (red) in brown adipocytes. Identical mitochondrial proteins found in both methods are in grey. MBC-flag pulldown proteins were affinity purified, gel resolved, excised, digested with trypsin, and analyzed with reverse-phase liquid chromatography with tandem mass spectrometry (LC-MS/MS). MBC-TurboID proximity labeling proteins were affinity purified and subjected to tandem mass tag (TMT) proteomic quantification. Identified proteins were crossed referenced with MitoCarta 3.0 and assessed for identical proteins between methods. B. BCAA catabolic pathway map. Proteins identified in both MBC-flag pulldown and MBC-TurboID proteomic methods are shown in bold. Proteins detected in only one method (HIBCH, MBC-flag pulldown; HMGCL, MBC-flag pulldown; MUT, MBC-TurboID) are un-bolded. C. Gene ontology of biological processes from the 284 proteins detected in both MBC-flag pulldown and MBC-TurboID proteomic methods. Red bars represent BCAA pathways. Data are represented as fold enrichment. D. Gc1 (Slc25a22) mRNA levels in indicated brown adipocytes. N = 4 per group. Statistic: One-way ANOVA with Dunnett’s multiple comparison’s test. E. mRNA levels of genes in BCAA metabolic pathway in MBC-KO and control brown adipocytes. N = 3 per group. Statistic: two-way ANOVA with Šídák’s multiple comparisons test. F. Unlabeled (M+0) metabolites in brown adipocytes incubated with 15N-BCAA for 24 hours. Data are relative to control group. N = 3 per group. Statistic: one-way ANOVA with Dunnett’s multiple comparisons test. [file NIHMS1982366-supplement-3.pdf]

**A**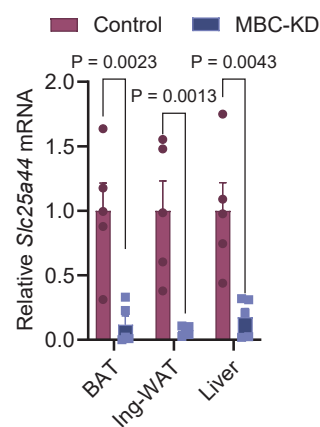**B**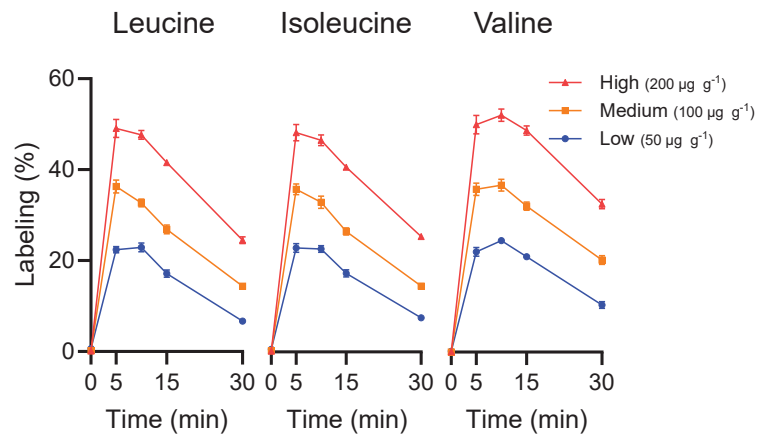**C**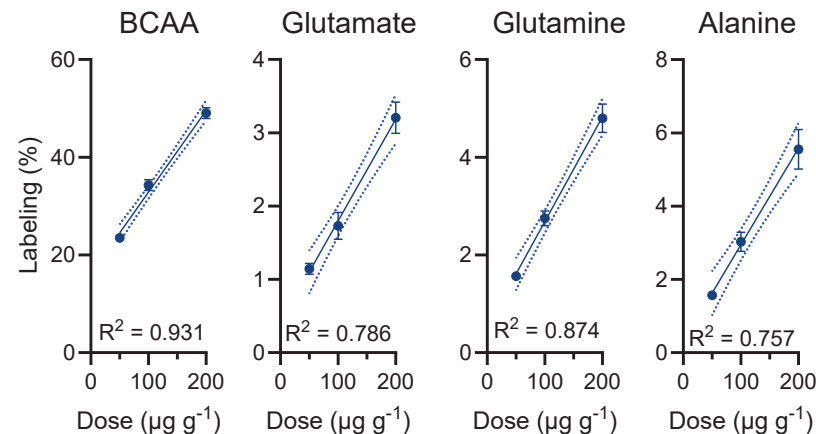**D**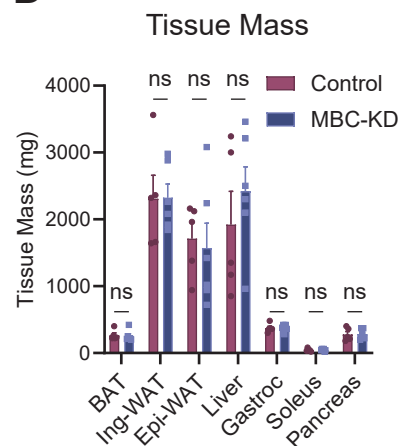**E**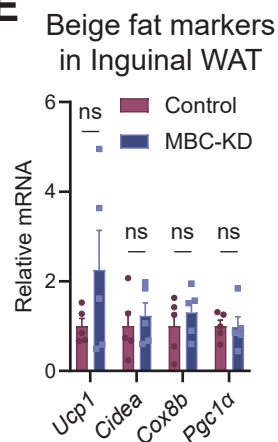**F**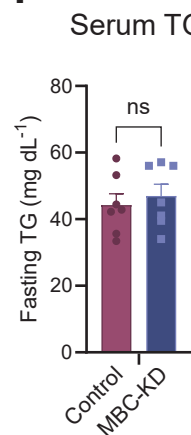**G**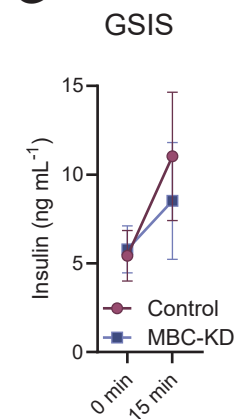**H**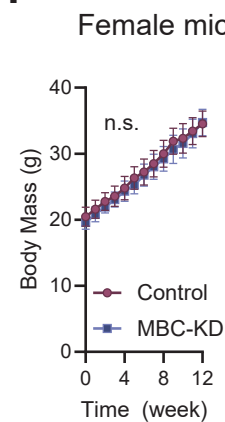**I**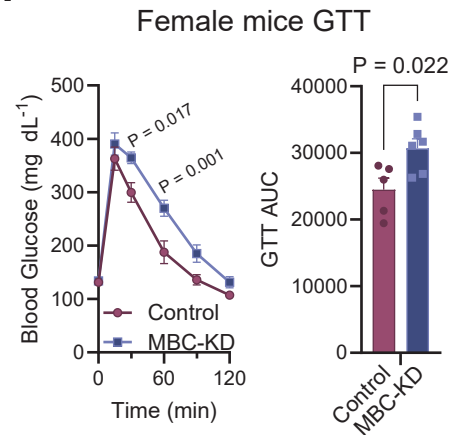**J**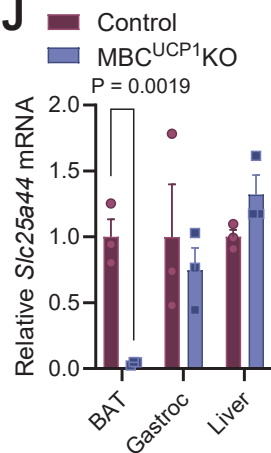**K**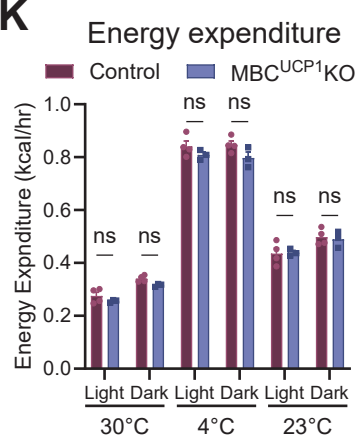**L**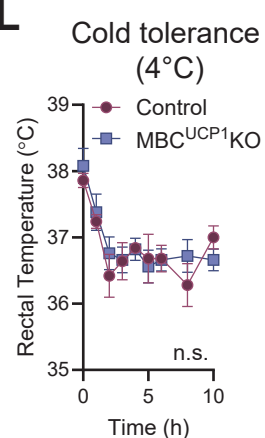**M**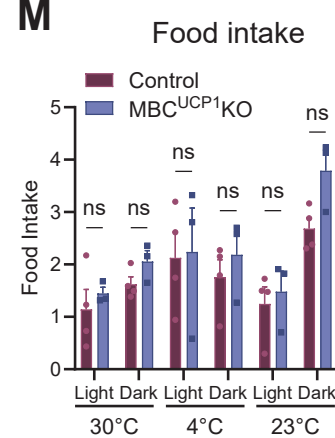**N**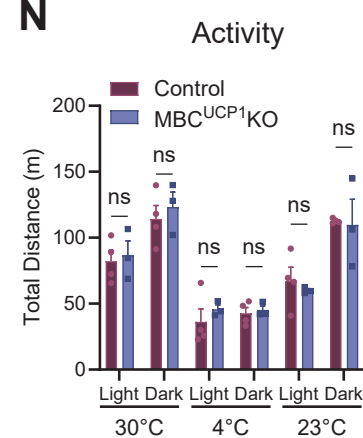**O**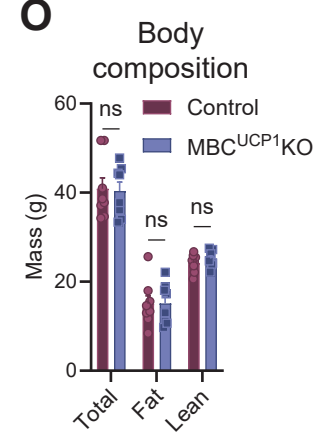

Supplement: 4 — A. Slc25a44 mRNA levels in indicated tissues from control and MBC-KD male mice. N = 5 per group per tissue. Statistic: unpaired t-test. B. Dose response of 15N-BCAAs in circulation in wild-type male mice over 30 minutes. Mice were given an intraperitoneal injection of BCAAs at a low (50 μg g−1 body mass), medium (100 μg g−1), or high (200 μg g−1) dose. N = 8 per dose. C. Mass action of BCAA and BCAA-derived metabolites in circulation 10 minutes after i.p. injection of 15NBCAA at a dose of 50, 100, or 200 μg per g body mass. N = 8 per dose, male mice. Statistic: coefficient of determination (R2). D. Tissue weights of high fat diet fed male control and MBC-KD, blue) male mice. N = 5 for control and 6 for MBC-KD. Statistic: unpaired t-test. E. Inguinal white adipose tissue mRNA of thermogenic markers from control and MBC-KD male mice. N = 5 per group. Statistic: unpaired t-test. F. Serum triglyceride (TG) levels in fasting control and MBC-KD male mice fed high fat diet. N = 7 per group. Statistic: unpaired t-test. G. Serum insulin levels in high fat diet fed control and MBC-KD male mice under fasting conditions (0 minutes) and 15 minutes after i.p. glucose delivery (glucose stimulated insulin secretion, GSIS). N = 7 per group. Statistic: 2-way ANOVA with Šídák’s multiple comparisons test. H. Body mass of female control and female MBC-KD mice on a high fat diet. N = 8 control and 11 MBC-KD. Statistic: multiple unpaired t-test with multiple comparisons corrected by two-stage step-up (Benjamini, Krieger, and Yekutieli) method. I. Glucose tolerance test and area under the curve (AUC) of high fat diet fed female control and female MBC-KD mice. Mice were fasted for 4 hours prior to collecting baseline blood glucose measurement and subsequent intraperitoneal injection of glucose (1 g kg−1 body mass). Glucose induced changes in blood glucose were recorded through 120 minutes post glucose delivery. N = 5 control mice and 6 for MBC-KD mice. Statistic for insulin tolerance curve [file NIHMS1982366-supplement-4.pdf]

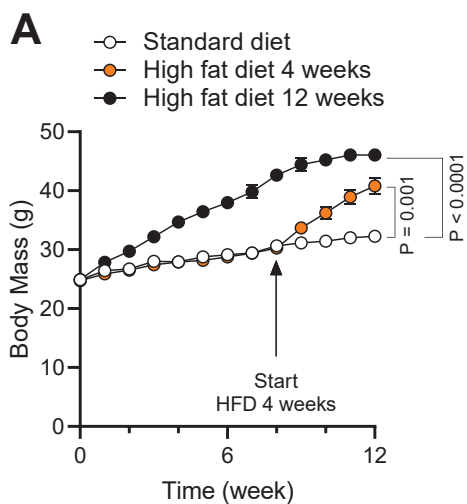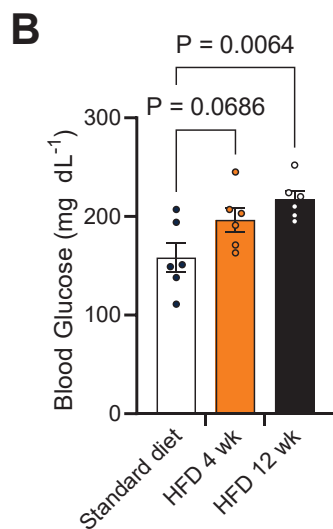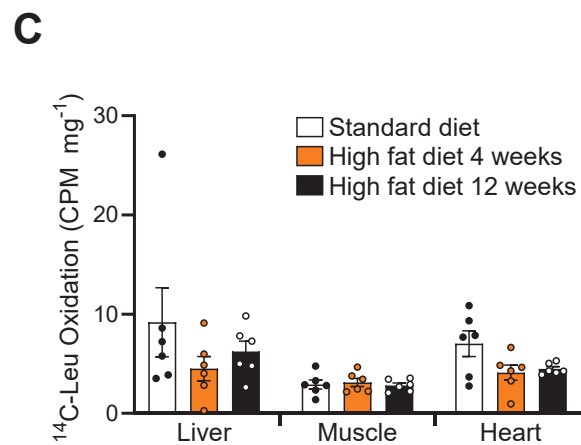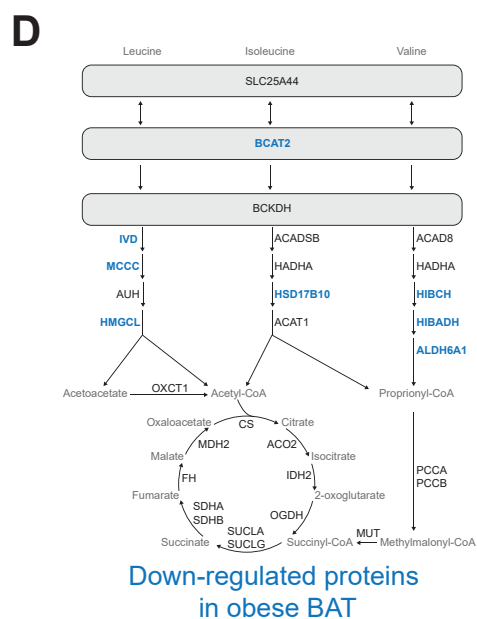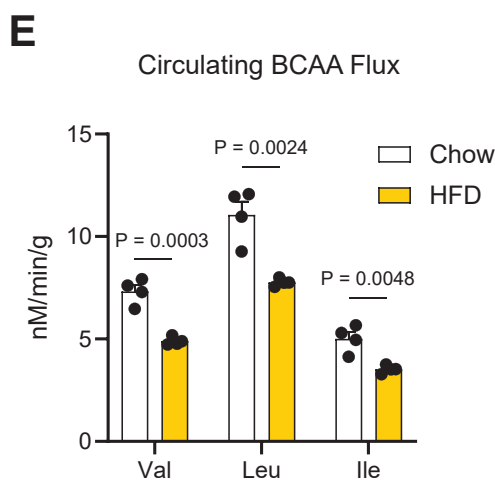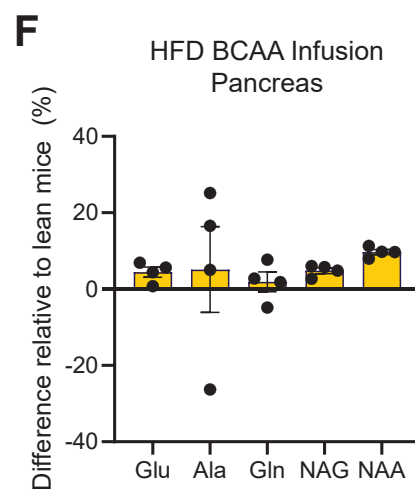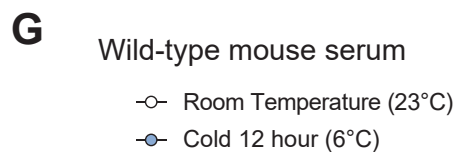

Supplement: 6 — A. Body mass of wild-type male mice fed a standard diet, short-term high fat diet (4 weeks), or long-term high fat diet (12 weeks). N = 6 per group. Statistic: 2-way ANOVA with Šídák’s multiple comparisons test. B. Fasting blood glucose of mice in (A). Statistic: One-way ANOVA with Dunnett’s multiple comparisons test. C. Ex vivo 14C-leucine oxidation in indicated tissues of mice (A). N = 6 per group. Statistic: One-way ANOVA with Dunnett’s multiple comparisons test. D. BCAA catabolic pathway map. Proteins identified as downregulated by high fat diet feeding, as shown in Figure 6B, are in blue bolded text. E. Circulating disposal rate (Rd, nmol min−1 g−1) of 15N-Leu, 15N-Val, and 15N-Ile in lean and obese mice. Male C57BL/6 mice with jugular vein catheters were fed a chow and high-fat diet for 8 weeks. A mixture of 15N-Leu, 15N-Ile, and 15N-Val in saline was infused via the catheter at a constant rate of 0.0836 μl/g/min. N = 4 per group. Statistic: unpaired t-test. F. Changes in indicated 15N-labeled metabolites in the pancreas of male mice fed a high-fat diet relative to mice fed a regular diet. Tissues were collected from mice stably infused with 15N-BCAA for 12 hours. N = 4 per group. Statistic: unpaired t-test. Data shows labelling percentage of tissue nitrogen metabolites normalized to the labeling percentage of tissue BCAA, expressed as the percent difference in obese mice relative to lean mice. G. Temperature induced changes in serum levels of BCAA-nitrogen derived metabolites N-acetylglutamate and glutathione. Serum was collected from wild-type male mice housed at room temperature (23°C). Serum was then collected after exposure to a 12-hour cold challenge at 6°C. N = 9 per group. Statistic: multiple paired t-test with two-stage step-up (Benjamini, Krieger, and Yekutieli) correction method. Line connects paired sample. [file NIHMS1982366-supplement-6.pdf]
