# Supplementary material for: BCAA nitrogen flux in brown fat controls metabolic health independent of thermogenesis
Source: Cell. Author manuscript; Available in PMC 2024 Jun 3. (PMC11145561; doi:10.1016/j.cell.2024.03.030)
Supplement: 5 — A. In vivo insulin stimulation of AKT Ser473 phosphorylation in indicated tissues. Mice were fasted 4 hours prior to insulin injection and rapid tissue excision. Western blots of phospho-AKTSer473 over total-AKT. N = 6 per group per tissue, male mice. B. Triglyceride content of livers from high fat diet fed control and MBCUCP1 KO mice. N = 6 per group. Statistic: unpaired t-test. C. Liver contents of indicated lipid species in control and MBCUCP1 KO male mice on a high-fat diet. N = 8 per group. Statistic: unpaired t-test. D. Western blot of lipid peroxidation marker 4-hydoxynonenal (4-HNE) from liver tissue of control and MBCUCP1 KO male mice fed high fat diet. N = 6 per group. E. Liver glutathione levels prior (left) and post supplementation of glutathione (right) in control and MBCUCP1 KO male mice. N = 6 per group prior and 7 for control and 6 for MBCUCP1 KO mice post. Statistic: unpaired t-test. F. Serum glutathione levels post-supplementation of glutathione in (A). Statistic: unpaired t-test. G. Glutathione levels in the serum (left) and liver (right) of wild-type male mice. Mice on a high-fat diet were supplemented with GSH (2g kg−1 d−1) or vehicle for 10 days. N = 10 for vehicle control and 9 for GSH supplementation. Statistic: unpaired t-test. H. Body weight changes in wild-type male mice in (C). Statistic is multiple unpaired t-test with multiple comparisons corrected by two-stage step-up (Benjamini, Krieger, and Yekutieli) method. I. Insulin tolerance test and area under the curve (AUC) of wild-type male mice supplemented with glutathione or vehicle. Mice were fasted for 4 hours prior to collecting baseline blood glucose measurement and subsequent intraperitoneal injection of insulin (0.8 U kg−1). N = 10 for vehicle control and 9 for GSH supplementation. Statistic for insulin tolerance curve is 2-way ANOVA with Šídák’s multiple comparisons test, and AUC statistic is unpaired t-test. J. In vivo insulin stimulation of AKTSer473 phosphorylation in liver of c [file NIHMS1982366-supplement-5.pdf]

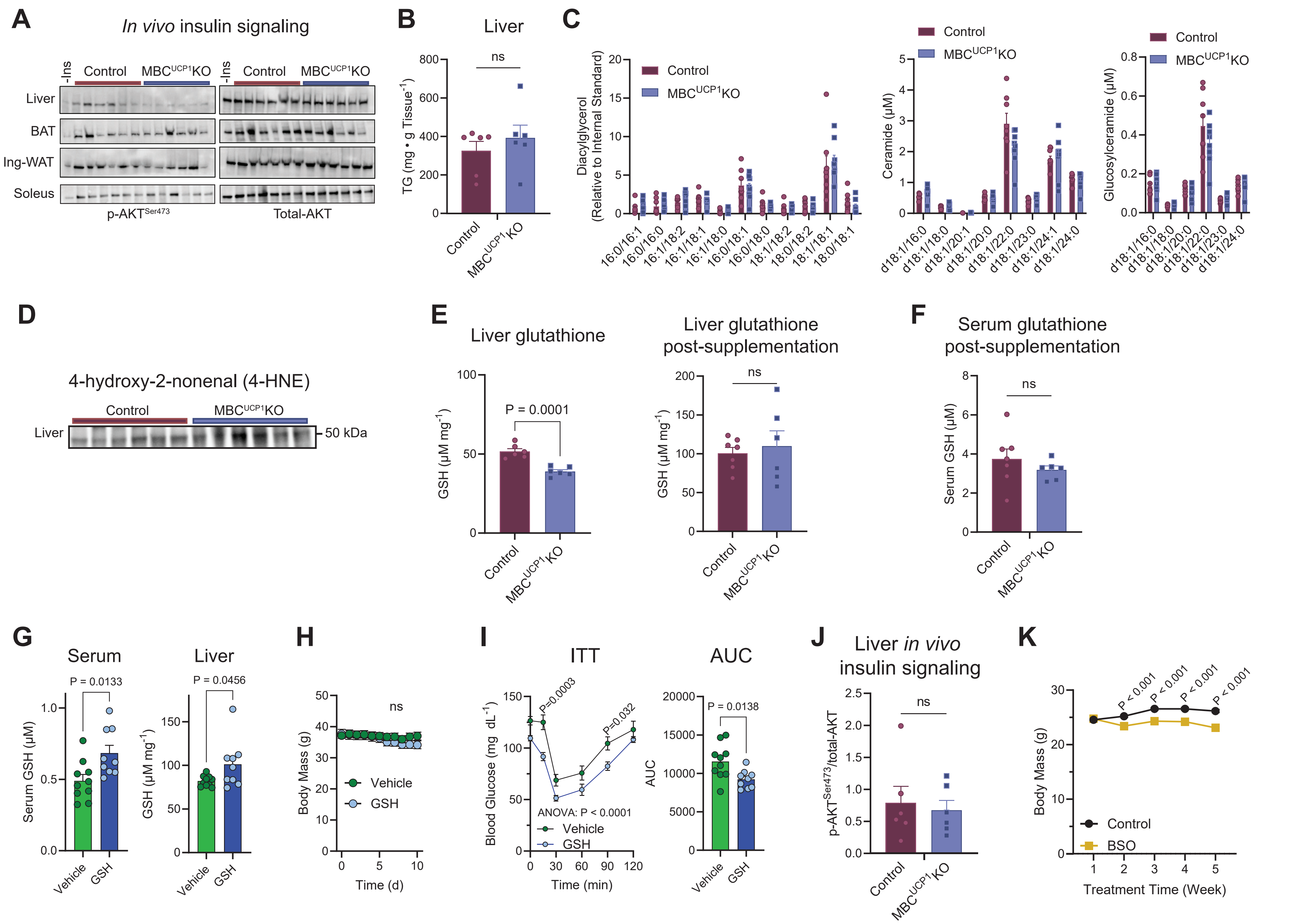

A
In vivo insulin signaling
-Ins
Control
MBCUCP1KO
Liver
BAT
Ing-WAT
Soleus
p-AKTSer473
Total-AKT
B
Liver
ns
TG (mg • g Tissue-1)
Control
MBCUCP1KO
C
Diacylglycerol (Relative to Internal Standard)
Ceramide (µM)
Glucosylceramide (µM)
16:0/16:1
16:0/16:0
16:1/18:2
16:1/18:1
16:1/18:0
16:0/18:1
16:0/18:0
18:1/18:2
18:0/18:2
18:1/18:1
18:0/18:1
d18:1/16:0
d18:1/18:0
d18:1/20:1
d18:1/20:0
d18:1/22:0
d18:1/23:0
d18:1/24:1
d18:1/24:0
D
4-hydroxy-2-nonenal (4-HNE)
50 kDa
E
Liver glutathione
P = 0.0001
GSH (µM mg-1)
Liver glutathione post-supplementation
F
Serum glutathione post-supplementation
Serum GSH (µM)
G
Serum
P = 0.0133
Liver
P = 0.0456
Vehicle
GSH
H
Body Mass (g)
Time (d)
I
ITT
P=0.0003
P=0.032
Blood Glucose (mg dL-1)
ANOVA: P < 0.0001
Time (min)
AUC
P = 0.0138
J
Liver in vivo insulin signaling
p-AKTSer473/total-AKT
K
P < 0.001
Body Mass (g)
BSO
Treatment Time (Week)
