# Supplementary material for: BCAA nitrogen flux in brown fat controls metabolic health independent of thermogenesis
Source: Cell. Author manuscript; Available in PMC 2024 Jun 3. (PMC11145561; doi:10.1016/j.cell.2024.03.030)
Supplement: 7 [file NIHMS1982366-supplement-7.pdf]

Table S1. MBC associated mitochondrial proteins detected by both FLAG-immunoprecipitation and Turbo-ID methods, related to Figure 3

MBC Proteome																			
1	Aars2	31	Aifm2	61	Coq10b	91	Etfa	121	Idh2	151	Mrpl9	181	Nfs1	211	Polrmt	241	Slc25a3	271	Tufm
2	Abcb10	32	Ak3	62	Coq3	92	Etfb	122	Idh3a	152	Mrps10	182	Nfu1	212	Ppox	242	Slc25a4	272	Uqcrc1
3	Abcb7	33	Ak4	63	Coq5	93	Etfdh	123	Idh3b	153	Mrps22	183	Nipsnap2	213	Pptc7	243	Slc25a40	273	Uqcrc2
4	Abcd3	34	Akap1	64	Coq6	94	Fars2	124	Idh3g	154	Mrps27	184	Noa1	214	Prdx3	244	Slc25a42	274	Uqcrfs1
5	Acaa1a	35	Aldh18a1	65	Coq8a	95	Fasn	125	Immt	155	Mrps28	185	Nt5dc2	215	Prodh	245	Slc25a44	275	Uqcrq
6	Acaa2	36	Aldh1l2	66	Coq9	96	Fastkd5	126	Ivd	156	Mrps30	186	Oat	216	Ptcd1	246	Slc25a5	276	Vars2
7	Acaca	37	Aldh2	67	Cox4i1	97	Fech	127	Lars2	157	Mrps31	187	Ogdh	217	Ptcd3	247	Spg7	277	Vdac1
8	Acacb	38	Aldh4a1	68	Cox5b	98	Foxred1	128	Letm1	158	Mrps35	188	Opa1	218	Ptges2	248	Sptlc2	278	Vdac2
9	Acad10	39	Aldh6a1	69	Cpt1a	99	Gatb	129	Lias	159	Mrps5	189	Osgepl1	219	Pycr1	249	Sqor	279	Vdac3
10	Acad8	40	Amacr	70	Cpt2	100	Gcdh	130	Lipt1	160	Mtch2	190	Oxa1l	220	Pycr2	250	Stoml2	280	Vwa8
11	Acad9	41	Apool	71	Crat	101	Gfm1	131	Lonp1	161	Mtg2	191	Oxct1	221	Rars2	251	Sucla2	281	Xpnpep3
12	Acadl	42	Atp5c1	72	Cs	102	Gfm2	132	Lrpprc	162	Mthfd1l	192	Pcca	222	Rdh13	252	Suclg1	282	Yars2
13	Acadm	43	Atpaf1	73	Cyp27a1	103	Glud1	133	Maip1	163	Mthfd2	193	Pccb	223	Rhot1	253	Suclg2	283	Yme1l1
14	Acads	44	Atpaf2	74	Dars2	104	Gpam	134	Mars2	164	Mtif2	194	Pck2	224	Rhot2	254	Supv3l1	284	Zadh2
15	Acadsb	45	Auh	75	Dbt	105	Gpd2	135	Mcat	165	Mto1	195	Pdha1	225	Rmdn3	255	Tamm41		
16	Acadvl	46	Bcat2	76	Decr1	106	Gpt2	136	Mccc1	166	Nadk2	196	Pdhb	226	Rpusd3	256	Tars2		
17	Acat1	47	Bckdha	77	Dhrs7b	107	Grpel1	137	Mccc2	167	Ndufa10	197	Pdhx	227	Rtn4ip1	257	Tbrg4		
18	Aco2	48	Bckdhb	78	Dhx30	108	Grsf1	138	Mcl1	168	Ndufa12	198	Pdk1	228	Samm50	258	Tefm		
19	Acot9	49	Bcs1l	79	Dlat	109	Gtpbp6	139	Mdh2	169	Ndufa4	199	Pdk4	229	Scp2	259	Tfam		
20	Acsf2	50	Bnip3	80	Dld	110	Guf1	140	Mgst1	170	Ndufa7	200	Pdpr	230	Sdha	260	Thnsl1		
21	Acsf3	51	Cat	81	Dlst	111	Hadha	141	Miga2	171	Ndufaf1	201	Pdss1	231	Sdhb	261	Timm23		
22	Acsl1	52	Cbr4	82	Dnaja3	112	Hadhb	142	Mipep	172	Ndufaf4	202	Pdss2	232	Sfxn1	262	Timm44		
23	Acsm3	53	Chchd6	83	Ech1	113	Hars2	143	Mocs1	173	Ndufaf7	203	Phb	233	Shmt2	263	Timm50		
24	Acss3	54	Cisd1	84	Echdc2	114	Hccs	144	Mpc2	174	Ndufb11	204	Phb2	234	Slc25a1	264	Tmlhe		
25	Adhfe1	55	Clpb	85	Echs1	115	Hibadh	145	Mpst	175	Ndufs1	205	Pitrm1	235	Slc25a10	265	Tomm20		
26	Afg1l	56	Clpx	86	Eci1	116	Hsd17b10	146	Mrpl12	176	Ndufs2	206	Pmpca	236	Slc25a11	266	Tomm40		
27	Afg3l1	57	Clybl	87	Eci2	117	Hsdl2	147	Mrpl19	177	Ndufs3	207	Pmpcb	237	Slc25a12	267	Trap1		
28	Afg3l2	58	Coa3	88	Ecsit	118	Hspa9	148	Mrpl2	178	Ndufs7	208	Pnpla8	238	Slc25a19	268	Trmt10c		
29	Agk	59	Coasy	89	Elac2	119	Hspd1	149	Mrpl44	179	Ndufv1	209	Pnpt1	239	Slc25a20	269	Trmt2b		
30	Aifm1	60	Comt	90	Eral1	120	Iars2	150	Mrpl45	180	Ndufv2	210	Poldip2	240	Slc25a22	270	Trmu		

Table S2. Subject characteristics of high and low BAT groups, related to Figure 6

	High BAT			Low BAT			*P*
Number of subjects	26			7			
Age (years)	23.2	±	0.64	23.0	±	1.18	0.889
Height (cm)	172.5	±	1.19	169.8	±	1.46	0.272
Weight (kg)	63.7	±	1.57	61.7	±	4.07	0.586
BMI (kg/m^2)	21.4	±	0.48	21.3	±	1.17	0.945
Body fat mass (kg)	11.2	±	0.78	9.4	±	1.70	0.307
Fat-free mass (kg)	52.5	±	1.06	52.3	±	2.64	0.927
Waist circumference (cm)	78.1	±	1.32	77.7	±	3.13	0.896
BAT activity (SUV)	7.46	±	1.03	0.71	±	0.09	0.002

Data represented as mean ± s.e.m. Statistic: unpaired t-test.

Table S3. Sequences of qPCR Primers, related to STAR Methods

Gene	Forward Primer	Reverse Primer
18S	AGTCCCTGCCCTTTGTACACA	CGATCCGAGGGCCTCACT
36B4	GGCCCTGCACTCTCGCTTTC	TGCCAGGACGCGCTTGT
Cidea	ATCACAACTGGCCTGGTTACG	TACTACCCGGTGTCCATTTCT
Cox8b	GAACCATGAAGCCAACGACT	GCGAAGTTCACAGTGGTTCC
Pgc1a	AGCCGTGACCACTGACAACGA	GCTGCATGGTTCTGAGTGCT
Gc1 (Slc25a22)	CATCGCTGGGCTAATCGGG	GCTCCCCTATACATGCCGAAG
Mbc (Slc25a44)	TCGCTGCTAACGTACATCCC	AGACAATGTGAGGGCACTCC
Ucp1	CACCTTCCCGCTGGACACT	CCCTAGGACACCTTTATACCTAATGG
Bcat1	ACATCAGAGCCTGGAAAGGTG	TATCTGGTTGTCCTTGCCGTA
Bcat2	TTCCAGAACCTCACGCTACAC	TAGCAGAACGTAGCATCCTGTC
Bckdhb	GCCAAAGACCCCACTGCAGTAA	GGATTTCCGCAATAGCTGTAGCACC
Dbt	CCAACATTGGATCAATCGGTGG	CTGTGATCCGCTGACCAGCTCAC
Dld	GCAGATCAACCAATTGAGGCTGACG	GCATGTTCCACCTAGTGTCTC
Ppm1k	GCAGCTCATTCGGTGACTGA	CCACTGGAGGCAAAGCTTCT
Bckdk	TGGTCATCACCATCGCCAAT	AGTGGTAGTCCATGACCCGA
Acad8	TCTGCATCGATCCTTCCTTG	CATTTCCCGAGCTGCAAAGT
Acads	GCACCAAAGCTTGGATCACC	GGAAGGCACTGATACCCTTGT
Acadsb	TATGCATCTGAGGTCGCTGG	CCGATCTTGGCATCTCGGAA
Acat1	ACCAGATGTGGTGGTGAAAGA	GGCAGCTGTTATTGTGCCATT
Acat2	AAGCCATGGGCAAGCTGAAA	CCATCGTTCATTCCTGATGCG
Aldh6a1	TCAGTGCCAACTGTAAAGCTC	TTCATTGGTGGCTGGGTTGT
Hadha	TGCTGACTGGCAGGAACATT	TTTATTCCTGGTCCCAGCGG
Hadhb	TTTCCCCACAGGCAGATTTCA	TTGGGAAGAACAGCTCAGAGG
Hibadh	ACCTTGGAATCAGGTCAGGC	GGGTTGTAAGTGTCGCTGGA
Hibch	AGGCGTCATAACGCTCAACA	AGGGTCTTGTTCCCATGTCTTT
Hmgcl	TGGGCTGCAGAATGAAAAGAGTA	GGGAGAAACAAAGCTGGTGG
Hsd17b10	TTTGCCCCAGCAAATGTGAC	TACCTGCACAGTTGACAGCC
Ivd	ATCACAGCCCCCGTTCAGTAT	AAGCCCGGGATATCTCTTCCA
Mccc1	TGGCAACGCAGTGTACTTGT	ATTAATACCAGGCGCTGGGG
Mccc2	TCCCAGTTTGCAGGTTACCA	CATGCATTCTACCCCCGACA
Mmut	TGATCCCCAAACACTGACCG	GCTTCAGGTAATGGGGCGAT
Oxct1	AACTACCGTGGTGGAGGTTG	TCTCCCTTTATGAGGCGGTG
Pcca	GCTGAGGACCCCTACAAGTC	CTGTCAACTCGGACACCAGG
Pccb	GCCAGCCGGTTTCAGTTAAAG	TGTTAGCTTCCCTCGCTTGT
